# Genome wide association study identifies four loci for early onset schizophrenia

**DOI:** 10.1038/s41398-021-01360-4

**Published:** 2021-04-27

**Authors:** Suqin Guo, Jiewei Liu, Wenqiang Li, Yongfeng Yang, Luxian Lv, Xiao Xiao, Ming Li, Fanglin Guan, Xiong-Jian Luo

**Affiliations:** 1 Henan Mental Hospital, The Second Affiliated Hospital of Xinxiang Medical University, Xinxiang, Henan 453002 China; 2 Henan Key Lab of Biological Psychiatry, International Joint Research Laboratory for Psychiatry and Neuroscience of Henan, Xinxiang Medical University, Xinxiang, Henan 453002 China; 3 Key Laboratory of Animal Models and Human Disease Mechanisms of the Chinese Academy of Sciences & Yunnan Province, Kunming Institute of Zoology, Chinese Academy of Sciences, Kunming, Yunnan 650223 China; 4 Department of Forensic Psychiatry, School of Medicine & Forensics, Xi’an Jiaotong University Health Science Center, Xi’an, Shaanxi 710061 China; 5 Center for Excellence in Animal Evolution and Genetics, Chinese Academy of Sciences, Kunming, 650223 China; 6 KIZ-CUHK Joint Laboratory of Bioresources and Molecular Research in Common Diseases, Kunming Institute of Zoology, Chinese Academy of Sciences, Kunming, Yunnan 650223 China

**Keywords:** Schizophrenia, Genetics

## Abstract

Early onset schizophrenia (EOS, defined as first onset of schizophrenia before age 18) is a rare form of schizophrenia (SCZ). Though genome-wide association studies (GWASs) have identified multiple risk variants for SCZ, most of the cases included in these GWASs were not stratified according to their first age at onset. To date, the genetic architecture of EOS remains largely unknown. To identify the risk variants and to uncover the genetic basis of EOS, we conducted a two-stage GWAS of EOS in populations of Han Chinese ancestry in this study. We first performed a GWAS using 1,256 EOS cases and 2,661 healthy controls (referred as discovery stage). The genetic variants with a *P* < 1.0 × 10^−04^ in discovery stage were replicated in an independent sample (903 EOS cases and 3,900 controls). We identified four genome-wide significant risk loci for EOS in the combined samples (2,159 EOS cases and 6,561 controls), including 1p36.22 (rs1801133, *P*_meta_ = 4.03 × 10^−15^), 1p31.1 (rs1281571, *P*_meta_ = 4.14 × 10^−08^), 3p21.31 (rs7626288, *P*_meta_ = 1.57 × 10^−09^), and 9q33.3 (rs592927, *P*_meta_ = 4.01 × 10^−11^). Polygenic risk scoring (PRS) analysis revealed substantial genetic overlap between EOS and SCZ. These discoveries shed light on the genetic basis of EOS. Further functional characterization of the identified risk variants and genes will help provide potential targets for therapeutics and diagnostics.

## Introduction

Schizophrenia (SCZ) is one of the severe chronic neurodevelopmental disorders with high heritability (approximately 80%)^1^. As a common mental disorder, SCZ affects ~0.5–1% global population^2^. The core symptoms of schizophrenia include positive symptoms (such as hallucinations and delusions), negative symptoms (social withdraw, anhedonia, alogia, avolition, lack of motivation), and cognitive impairments (SCZ patients have poor cognitive performance compared to normal individuals, including poor working memory and executive function)^3^. So far, the pathogenesis of SCZ remains largely unknown. Nevertheless, high heritability indicates the pivotal role of genetic factors in SCZ. To uncover the risk genes and dissect the genetic architecture of SCZ, multiple genome-wide association studies (GWASs) have been performed in world populations and over 200 SCZ risk loci have been identified^4–18^. The onset age of most SCZ cases is usually in late adolescence or early adulthood^19,20^. However, about 5% SCZ cases has an illness onset before age 18, which is usually defined as early onset schizophrenia (EOS)^21^. As a rare and severe form of SCZ, EOS cases have increased disease severity, worse treatment outcome, and prognosis compared with adulthood SCZ patients^22–24^.

Accumulating evidence suggest that studying EOS is of great value in elucidating the genetic architecture and pathogenesis of SCZ^25,26^. Compared with the late or adult onset cases, EOS cases have more salient genetic (or familial) risk factors (i.e., genetic risk factors play a more salient role in EOS compared with late or adult onset cases, which leads to early manifestation of symptoms and illness onset of EOS cases) (including a higher rate of cytogenetic abnormalities) and more severe neurodevelopmental anomalies^26–28^. Despite the fact that genetic study of EOS may help identify important risk genes for SCZ, unfortunately, there is no systematic GWAS of EOS been reported to date. Due to the low prevalence of EOS, recruitment of adequate sample size of EOS cases is quite difficult. Accordingly, conducting GWAS of EOS is challenging in psychiatric genetics. To identify the risk variants for EOS and to uncover its genetic basis of EOS, we carried out a two-stage genetic analysis of EOS in this study. In the discovery stage, we conducted a GWAS using 1,256 EOS cases and 2,661 healthy controls. The single-nucleotide polymorphisms (SNPs) with *P* values less than 1.0 × 10^−04^ in the discovery stage were then replicated in an independent sample (903 EOS cases and 3,900 controls). Our meta-analysis identified four risk loci for EOS (including 1p36.22 (rs1801133), 1p31.1 (rs1281571), 3p21.31 (rs7626288), and 9q33.3 (rs592927)). Our study reports the first genome-wide significant (GWS) risk variants for EOS and provides new insights into its genetic architecture of EOS. Future functional investigation of these risk variants and genes will facilitate biomarker identification and drug development.

## Methods

### EOS cases recruitment and samples in the discovery stage

In the discovery stage, 1,442 EOS cases and 4,027 controls were recruited. All participants were Han Chinese. EOS cases were recruited from October 2010 to September 2018 at the Henan Medical Health Center (The Second Affiliated Hospital of Xinxiang Medical University). EOS cases were assessed with Structured Clinical Interview for DSM-IV Axis I Disorders (SCID) by experienced psychiatrists and SCID raters, and diagnosis was made based on the Diagnostic and Statistical Manual of Mental Disorders IV (DSM-IV) criteria. Detailed information about the age at onset, symptoms, clinical course, treatment history, medical records, and family history of psychiatric disorders were collected and evaluated. The age at first manifestation of positive symptoms^29^ was defined as the age at onset of SCZ. The age at onset information was derived from the Comprehensive Assessment of Symptoms and History (CASH)^30^. Standard diagnostic assessments were supplemented with interviews of family informants and careful evaluations of the detailed clinical information. All available information was used to reach a consensus DSM-IV diagnosis by at least two experienced psychiatrists. EOS cases with a history of drug abuse and head injury were excluded in this study. In addition, cases with neurological diseases (including multiple sclerosis and epilepsy), pervasive developmental disorder (including mental retardation), and other psychiatric disorders (including autism, bipolar disorder, depression, and attention deficit hyperactivity disorder) were also excluded. Controls with a family history of psychiatric disorders were excluded. Informed consents were obtained from all participants (including subjects included in the discovery and replication stages, and informed consents were provided by parents or guardians when the EOS cases could not provide valid informed consent). This study was approved by the internal review boards of the Second Affiliated Hospital of Xinxiang Medical University and the Kunming Institute of Zoology.

The EOS cases in the replication stage were recruited from the city of Xi’an in Shaanxi province. All SCZ patients were EOS, which was defined in the study as onset before 18 years as first manifestation of SCZ symptoms. All patients were recruited at the Xi’an Mental Health Center based on the following criteria: (a) receiving diagnosis strictly according to the DSM-IV by at least two experienced psychiatrists; (b) having ages at onset before 18 years; (c) receiving antipsychotic treatment and maintaining a stable condition; (d) not being first-episode SCZ given that initial diagnoses are often unreliable; and (e) having no substance-induced psychotic disorders, learning disabilities, head injuries, or other symptomatic psychoses except SCZ. The study was approved by the Medical Ethics Committee of Xi’an Jiaotong University. The diagnosis criteria of EOS cases were the same as those for the EOS cases in the discovery stage. All participants (or their parents/legal guardians) provided informed consents and this study was approved by the internal review board of Xi’an Jiaotong University. Of note, the EOS cases from the discovery and replication stages were recruited from Xinxiang medical university and Xi’an Jiaotong university independently.

After strict quality control (QC) (see below), a total of 1,256 EOS and 2,661 controls were retained. The mean onset age of the included EOS cases was 14.57 ± 2.27 years old. 41% cases were males and 59% cases were females. All controls (55% were males and 45% were females) were healthy volunteers with a mean age of 28.60 ± 7.01 years (Supplementary Table S1). This study was approved by the internal review boards of the Second Affiliated Hospital of Xinxiang Medical University and the Kunming Institute of Zoology.

### EOS cases and controls in the replication stage

In the replication stage, we recruited 1,001 EOS cases and 4,068 healthy controls. The detailed information about replicated samples are provided in the above part. After stringent QC (see below), a total of 903 EOS and 3,900 controls were retained. The mean onset ages of EOS cases (of which 36% were males and 64% were females) in replication stage was 14.39 ± 1.94 years old. And the average age of controls (of which 54% were males and 46% were females) was 28.55 ± 6.51 years old (Supplementary Table S1). All participants (or their parents/legal guardians) provided informed consents and this study was approved by the internal review board of Xi’an Jiaotong University.

### DNA extraction and genotyping

DNA extraction and genotyping procedures are provided in Supplementary Material.

### Quality control

We first performed individual-level QC as follows: (1) we performed sex check and subjects with inconsistent results were excluded. We also excluded samples whose sex could not be accurately estimated based on the genotype data; (2) we calculated the missing rate and samples with missing rate >3% were excluded; (3) we calculated heterozygosity of each sample and outliers were removed if it located 3 standard deviation (s.d.) away from the mean of heterozygosity of all samples; (4) we removed genetically related samples using KING software (http://people.virginia.edu/~wc9c/KING/)^31^. We used --related flag to detect the potential kinship coefficients that were within third degree. We also used the GCTA software^32^ to double check the cryptic relations among included subjects with following command: –grm-cutoff 0.025; (5) we conducted principal component analysis (PCA) and outliers were excluded. EIGENSOFT smartpca program (https://www.hsph.harvard.edu/alkes-price/software/)^33,34^ and GCTA^32^ were used for PCA. For PCA, we downloaded resequencing data from the 1000 Genomes project including YRI (*N* = 108), CEU (*N* = 99), CHB (*N* = 103), CHS (*N* = 105), JPT (*N* = 104)^35^. We firstly performed PCA using the resequencing data from the 1000 Genomes project and genotypes of our cases and controls. We then carried out PCA analysis in our EOS cases and controls to exclude outliers (MHC region (hg 19, chr6:25MB-34MB) was excluded in PCA). We calculated top 20 PCs for each iteration, samples with a >6 s.d. away from the mean of each PCs were excluded. We performed five iterations in total. Among the PCs calculated for subgroup 1 and subgroup 2, we evaluated the effect of different PCs on the result of GWAS summary statistics to determine the PC inclusion in our final GWAS result. Based on this, we finally selected 16 PCs and 6 PCs for subgroup 1 and subgroup 2 as covariates, respectively.

We then conducted variant-level QC as follows: (1) SNPs with a genotyping call rate ≥97% were retained; (2) SNPs that were significantly deviated from Hardy–Weinberg equilibrium (HWE) in controls (HWE *P* < 1 × 10^−06^) and cases (HWE *P* < 1 × 10^−10^) were excluded; (3) only SNPs with a minor allele frequency (MAF) > 0.01 were retained; (4) we only analyzed biallelic SNPs in this study. After strict QC, 447,334 variants were remained for the discovery stage. The major QC step was performed by plink (version 1.9) software^36^.

### Imputation

Imputation was performed using minimac3 software^37^, and the 1000 Genomes project phase 3 data (which were downloaded from the minimac3 website (https://genome.sph.umich.edu/wiki/Minimac3)) was used as the reference panel. Before imputation analysis, we firstly utilized Eagle to do phase analysis^38^, imputation was then implemented on the phased genotype data. The QC of the imputed genotype data were as follows: (1) imputation quality score >0.8 in discovery stage and >0.6 in replication stage; (2) MAF > 0.01; (3) HWE QC parameters were same as the discovery stage; (4) only biallelic SNPs were remained for further association test. As the genotyping platforms of our replication study were different (which prohibited us to perform a GWAS in the replication stage), to include more variants in the replication stage, we used a slightly relax criterion for the QC of the imputation. Of note, this is a balance between imputation QC and the number of SNPs included in the replication analysis. The flowchart of our QC steps are provided in Supplementary Fig. S1.

### Genome-wide association analysis

Genetic association analysis was carried out by using logistic regression analysis (implemented in PLINK (version 1.9)^36^), adjusting for the significant PCs (top 16 PCs for subgroup 1 and top 6 PCs for subgroup 2) and sex (as covariates) of our cases and controls. The Manhattan and QQ plot were plotted by using CMplot R package (https://github.com/YinLiLin/R-CMplot) in R environment (version 3.5.0).

### Meta-analysis

Replication EOS cases (*N* = 1,001) and controls (*N* = 4,068) were genotyped with the Illumina ASA and GSA SNP array platforms, respectively. QC were performed following the same steps and criteria in the discovery stage. After strict QC, 903 EOS case and 3,900 controls were retained. A total of 439,797 (for ASA) and 354,150 (for GSA) biallelic SNPs passed QC, and only 133,620 SNPs overlapping in GSA and ASA arrays were observed. Imputation was performed separately. SNPs with *P* value less than 1.0 × 10^−04^ in the discovery stage were analyzed in the replication samples and meta-analysis (fixed-effect model) was performed using PLINK (version 1.9)^36^. The GWS loci were visualized with the Locuszoom tool (http://locuszoom.org/)^39^.

### Brain expression quantitative trait loci (eQTL) analysis and differential expression analysis in schizophrenia cases and controls

To explore if the identified variants were associated with gene expression in human brain tissues, we examined the associations between the identified risk variants and gene expression using two independent brain eQTL datasets, the Common Mind Consortium (CMC)^40^, and LIBD dataset^41^. We further examined the expression level of putative target genes of the identified GWS variants in SCZ cases and controls using expression data from the PsychEncode^42^. Detailed information on the eQTL and differential expression analyses are provided in Supplementary material.

### Polygenic risk scoring (PRS) analysis

To investigate if the published GWAS summary statistics of SCZ were associated with the case–control status of our EOS samples, we conducted PRS analysis using PRSice-2.0 software^43^. The information about training datasets and PRS analysis are provided in Supplementary material.

### LD score regression analysis

LD score regression analysis procedures are provided in the Supplementary Material.

### Conservation analysis and 3D structure modeling

The multiple alignments of the protein sequence containing rs1801133 in diverse species were generated using UCSC genome browser (https://genome.ucsc.edu/) and MEGA software^44^. We downloaded the DNA sequence (in fasta format) and then performed multiple alignments using MEGA software after translating the coding sequences into protein sequences. The 3D structure of MTHFR proteins with Ala and Val at rs1801133 site were modeled with Swissmodel (https://swissmodel.expasy.org/). These two protein structures were modeled based on a known protein structure (PDB id: 6FCX, http://www.rcsb.org/structure/6FCX)^45^. Risk loci were defined as described in study of PGC2^14^.

### Tissue and GO/KEGG enrichment analysis by MAGMA

MAGMA^46^ analysis procedures are provided in the Supplementary Material.

## Results

### Identification of two genetically matched subgroups in the discovery samples

In the discovery stage, we conducted a GWAS meta-analysis in 1,256 EOS and 2,661 healthy controls. After strict QC and imputation (detailed filter procedures were described in the “Methods” section), a total of 4,867,007 SNPs were retained for genome-wide association (GWA) analysis. We firstly performed principal component analysis (PCA) using the genotypes of individuals from the 1000 Genomes project^35^ (a total of 99 Europeans (CEU), 103 Northern Han Chinese (CHB), 105 Southern Han Chinese (CHS), 104 Japanese (JPT), 108 Africans (YRI)) and the genotypes of our sample. PCA showed that all the cases and controls were of Han Chinese ancestry (Supplementary Fig. S2). We further conducted PCA in our cases and controls to explore if there was potential population stratification. The PCA result revealed two clusters (subgroups), which may reflect subtle population structure in our sample^47^ (Supplementary Fig. S3). We thus divided our sample into two genetically matched case–control subgroups (based on the PCA). The first subgroup (referred as subgroup 1) contained 860 EOS cases and 1,505 controls. The second subgroup (referred as subgroup 2) included 396 EOS cases and 1,156 controls. The genomic inflation factor (*λ*_GC_) of the first and second subgroups were 1.06 (excluded MHC (chr6:25-34MB) and adjusted for top 16 PCs) and 1.01 (excluded MHC and adjusted for top 6 PCs), respectively. As *λ*_GC_ of subgroup 1 showed tiny inflation, we performed LDSC analysis to test whether this inflation was due to polygenicity or confounding effect^48^. LDSC analysis showed that the intercept of subgroup 1 was 1.03, which is very close to 1, indicating that the polygenicity other than confounding led to the tiny *λ*_GC_ inflation of subgroup 1. Therefore, the observed significant associations were unlikely attributed to population stratifications in these two genetically matched groups (Supplementary Figs. S4–S7).

### Identification of four GWS risk loci for EOS

**Fig. 1:**
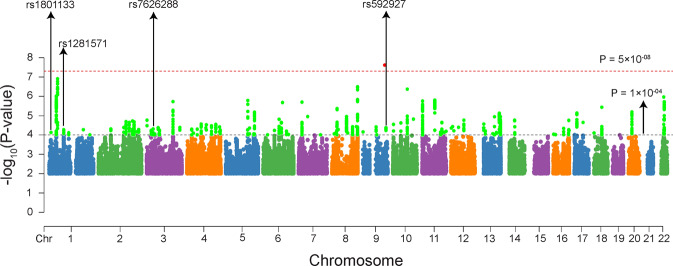
Manhattan plot of EOS GWAS associations. Associations were from the GWAS meta-analysis of the discovery stage (*N* = 1,256 EOS cases and 2,661 controls). The dashed black horizontal line indicates the suggestive *P* threshold (*P* < 1.0 × 10^−04^). The genome-wide significant *P* threshold (*P* < 5.0 × 10^−08^) is represented as the dashed red horizontal line. The arrows indicate the four genome-wide significant SNPs in our combined discovery and replication samples.

We carried out GWA analysis in the first and second subgroups separately. The quantile–quantile plots for these two GWA analyses are presented in the Supplementary Figs. S8 and S9. We then performed a meta-analysis through combining the GWA results from the two subgroups (Fig. 1 and Supplementary Fig. S10). Our meta-analysis identified 1,061 SNPs that showed suggestive associations with EOS (*P* < 1.0 × 10^−04^), suggesting that these SNPs may be associated with EOS.

**Fig. 2:**
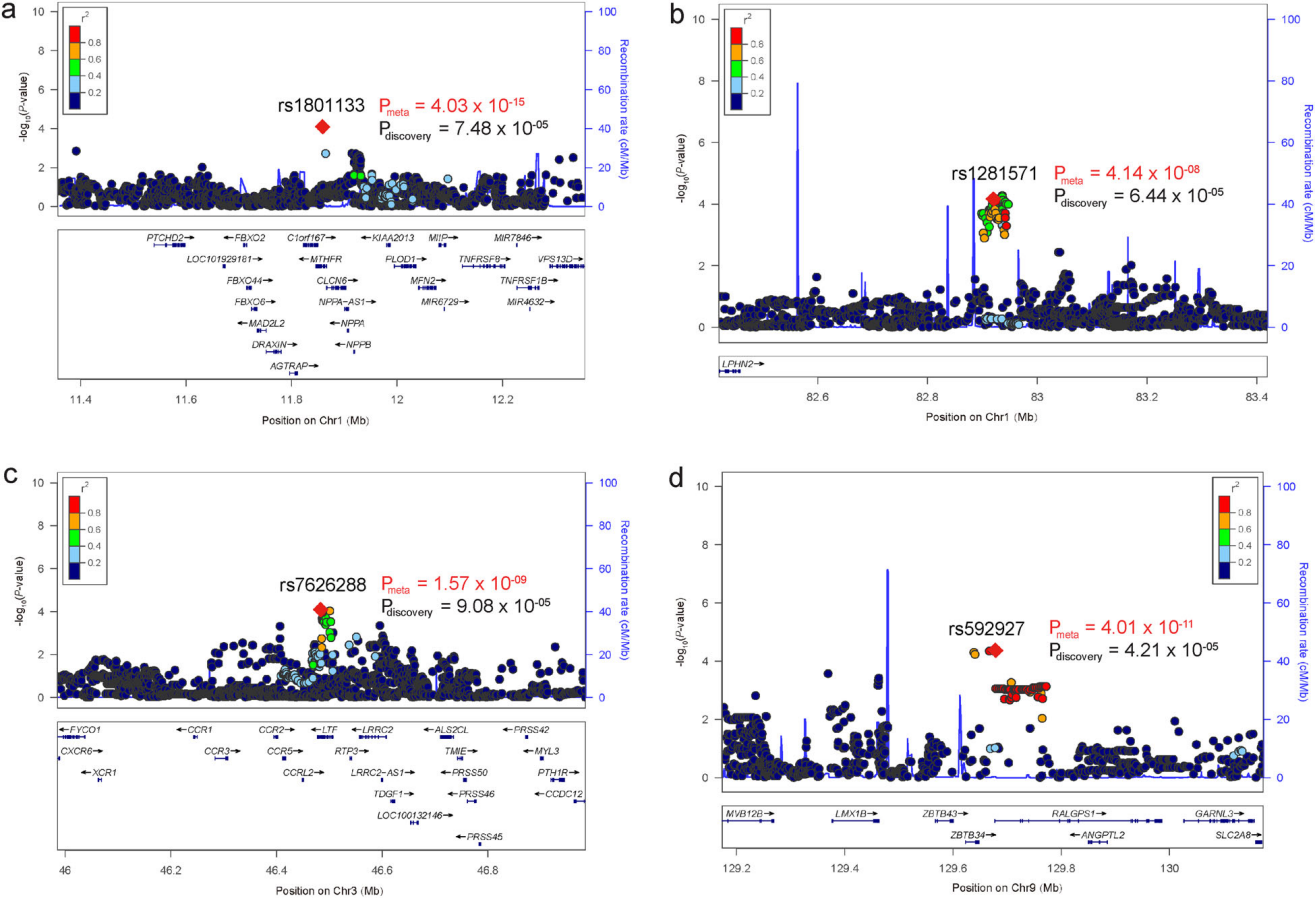
Locuszoom plots of the genome-wide significant (LD independent) risk variants. **a** rs1801133 is located in the fifth exon of *MTHFR*. **b** rs1281571 is located in the downstream of *LPHN2*. **c** rs7626288 is located in the intron 12 of *LTF*. **d** rs592927 is located in the upstream of *RALGPS1*.

To further validate our results, we replicated the SNPs with *P* values less than 1.0 × 10^−04^ in an independent EOS sample (including 903 cases and 3,900 controls). Through meta-analyzing (fixed-effect model was used) the association results from the discovery and replication stages (a total of 2,159 EOS cases and 6,561 controls), we identified four GWS loci for EOS (Table 1 and Fig. 2). We also listed the SNPs that showed suggestive association with EOS (*P* < 1.0 × 10^−04^) in Supplementary Table S2.

**Table 1:** Genome-wide significant loci identified in this study.

Chr^a^	Position	Index SNP	A1/A2	Discovery stage (1256 cases/2661 controls)			Replication stage (903 cases/3900 controls)			Meta-analysis (2159 cases/6561 controls)		
				*P*	OR^b^	AF^c^	*P*	OR	AF	*P*^d^	OR	*I*^e^
1	11856378	rs1801133	A/G	7.48 × 10^−05^	1.24	0.59/0.50	1.20 × 10^−12^	1.45	0.54/0.45	4.03 × 10^−15^	1.35	82.65
1	82919925	rs1281571	A/G	6.44 × 10^−05^	1.24	0.44/0.40	1.70 × 10^−04^	1.22	0.45/0.40	4.14 × 10^−08^	1.23	0
3	46485369	rs7626288	A/G	9.08 × 10^−05^	0.81	0.39/0.43	3.63 × 10^−06^	0.78	0.38/0.44	1.57 × 10^−09^	0.80	0
9	129673226	rs592927	A/G	4.21 × 10^−05^	1.30	0.24/0.20	1.56 × 10^−07^	1.38	0.25/0.19	4.01 × 10^−11^	1.34	0

^a^Chromosome. ^b^OR: odds ratio (based on allele A1). ^c^AF: allele frequency (case AF/control AF, based on allele A1). ^d^Fixed-effect meta-analysis *P* value. ^e^*I*^2^ heterogeneity index of meta-analysis of discovery stage ASA group 1, group 2, and replication stage samples.

**Fig. 3:**
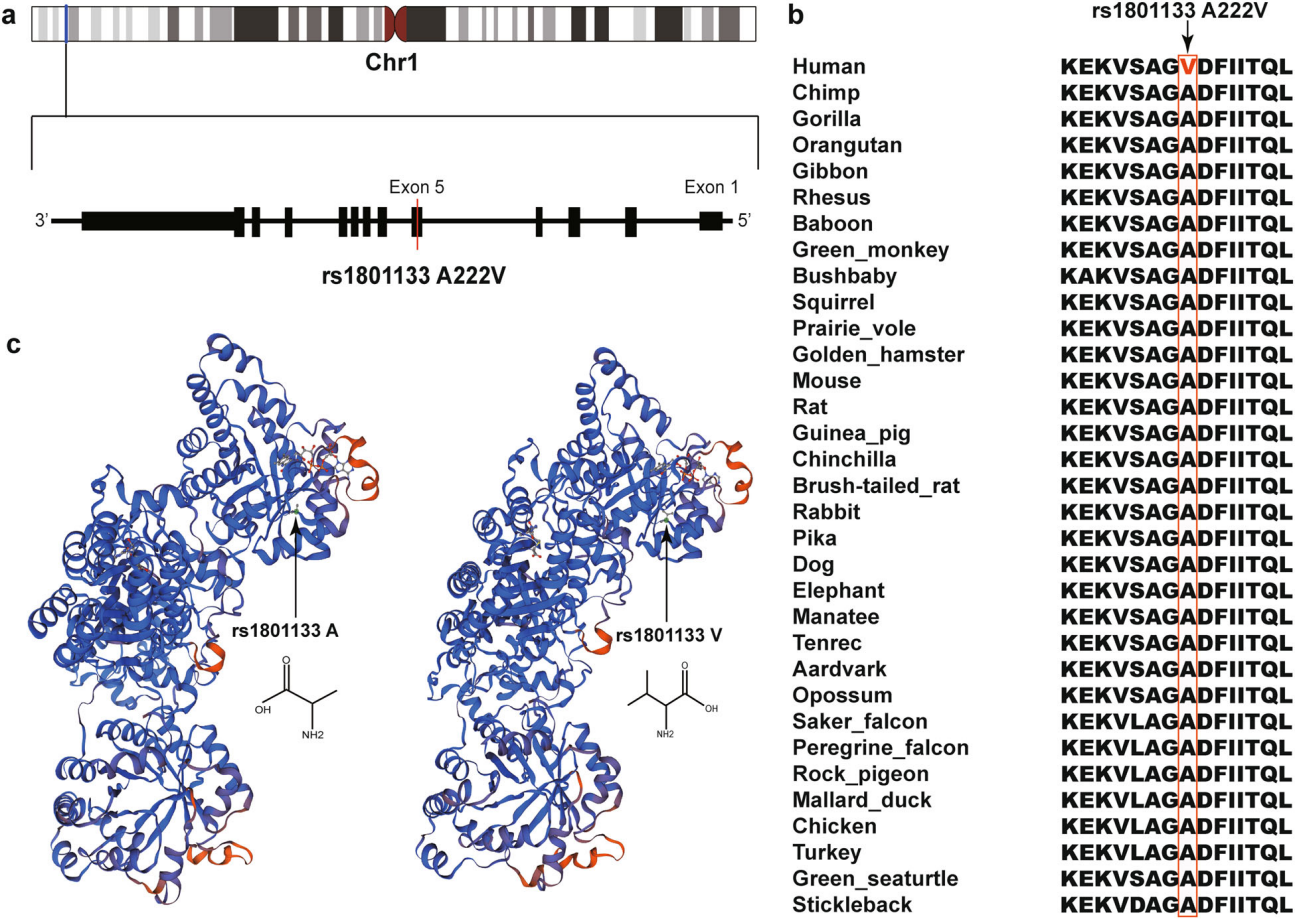
Genomic location of rs1801133 and the 3D structure of MTHFR. **a** rs1801133 is located in the fifth exon of *MTHFR*. **b** rs1801133 is located in an evolutionary highly conserved genomic region. The amino acid encoded by rs1801133 was Ala in most of the species. However, a new amino acid (Val) has emerged in humans. **c** The 3D structure of MTHFR with different amino acids at rs1801133.

The first GWS locus was at 1p36.22 (rs1801133) (Fig. 2a). The most significant SNP in this locus was a non-synonymous SNP rs1801133, which located in the coding region (exon 5, pA222V) of *MTHFR* (Fig. 3a). The ancestry allele (G allele, protective allele) of rs1801133 encodes alanine (Ala) while the derived allele (A allele) encodes valine (Val).

*MTHFR* encodes methylenetetrahydrofolate reductase, which converts 5,10-methylenetetrahydrofolate into 5-methyltetrahydrofolate. We noticed that rs1801133 was located in a highly evolutionarily conserved region (Fig. 3b), suggesting the functional importance of this region. Consistently, previous studies have shown that rs1801133 was a functional variant, and the activity of MTHFR protein with Val at rs1801133 was reduced compared with the Ala^49^. These data suggest that rs1801133 may confer risk of EOS through affecting MTHFR activity. The second GWS variant is rs1281571, was located in the downstream region of *LPHN2* (Fig. 2b). The third GWS variant is rs7626288 was located in the intron 12 of *LTF* gene (Fig. 2c). And the fourth GWS variant rs592927 was located in the upstream of *RALGPS1* (Fig. 2d).

### Identification of potential target genes of the GWS variants

To explore the potential effects of the identified GWS variants, we examined the associations between these variants and gene expression in the human brains using the eQTL data from the CMC^40^ and LIBD^41^. In addition to affecting MTHFR enzymatic activity, eQTL analysis showed that rs1801133 was also associated with the expression of *MFN2* (*P* = 2.69 × 10^−03^) and *MTHFR* (*P* = 5.83 × 10^−03^) in the CMC eQTL dataset, with risk allele (A allele) associated with higher *MFN2* and lower *MTHFR* expression level (Supplementary Fig. S11). Besides, we found that rs1801133 was associated with *NPPA-AS1* expression in the LIBD eQTL dataset (*P* = 3.63 × 10^−06^). The GWS SNP rs7626288 was an eQTL of *TDGF1* (*P* = 5.67 × 10^−05^) in the LIBD dataset. And in the CMC dataset, rs592927 was associated with *ANGPTL2* (*P* = 2.99 × 10^−3^) and *RALGPS1* expression (*P* = 4.01 × 10^−6^) (Supplementary Fig. S11 and Table S3). These eQTL results suggested that the GWS variants might confer risk of EOS by regulating the expression of these eQTL genes.

We also explored if the SNPs associated with *MTHFR* (i.e., eQTL)^40^ or methylation (methylation quantitative trait loci, meQTLs)^50^ are also associated with EOS. In the CMC dataset, 214 SNPs showed associations with *MTHFR* expression. Among them, five SNPs, including rs1801133, rs2981953, rs12402363, rs12406383, and rs198369, were also showed nominal associations (*P* < 0.05) with EOS. In addition, 93 SNPs were associated with methylation of *MTHFR*. Among them, eight SNPs, including rs1801133, rs2981953, rs198389, rs198379, rs549596, rs198388, rs198369, and rs198358 were nominally associated with EOS (Supplementary Table S4). These results suggest that these variants might confer risk of EOS by modulating *MTHFR*. However, more work are needed to validate this.

### Dysregulation of potential target genes of the GWS variants in schizophrenia cases compared with controls

Our eQTL analysis suggested that the GWS variants might confer risk of EOS by regulating the expression level of the potential target genes (i.e., genes whose expression level were associated with the GWS variants). To further explore if the expression levels of the potential target genes were dysregulated in SCZ cases compared with controls, we examined the expression of the potential target genes using RNA-seq-based expression data from the PsychEncode (including 559 SCZ cases and 936 controls)^42^. Interestingly, we found that *MTHFR* (*P* = 9.70 × 10^−04^), *TDGF1* (*P* = 2.33 × 10^−04^), and *ANGPTL2* (*P* = 1.46 × 10^−05^) were significantly down-regulated in brains of SCZ cases compared with controls. In addition, we also noticed that *RALGPS1* showed a trend of down-regulation in SCZ cases compared with controls (*P* = 0.079). These expression data indicated the dysregulation of these genes in SCZ, suggesting that GWS variants might contribute to SCZ susceptibility by regulating the expression of these genes.

### Polygenic risk score profiling

Although EOS is a rare form of SCZ, we speculated that EOS might share genetic basis with the SCZ. Accordingly, the GWAS results of SCZ could be used to calculate the polygenic burden of each EOS sample and associate it with disease status. We performed PRS analyses (using PRSice-2 software)^43^ to explore if the PRS score is associated with the case–control status of our sample. The genotype data of our samples in the discovery stage were used as the target sample. We used summary statistics from three large-scale GWASs of SCZ as training sets. The first training set was from the study by Pardinas et al.^9^ (referred as CLOZUK + PGC2). The second training set was from a large-scale meta-analysis of Chinese samples and PGC2 samples^7^ (referred as Chinese +PGC2). The third training set was a recent study and the summary statistics of East Asian ancestry was used (referred as EAS)^6^. We set 10 *P* value thresholds (*P*_T_), including 5.00 × 10^−08^, 5.00 × 10^−05^, 1.00 × 10^−04^, 0.001, 0.01, 0.05, 0.1, 0.2, 0.5, and 1.

**Fig. 4:**
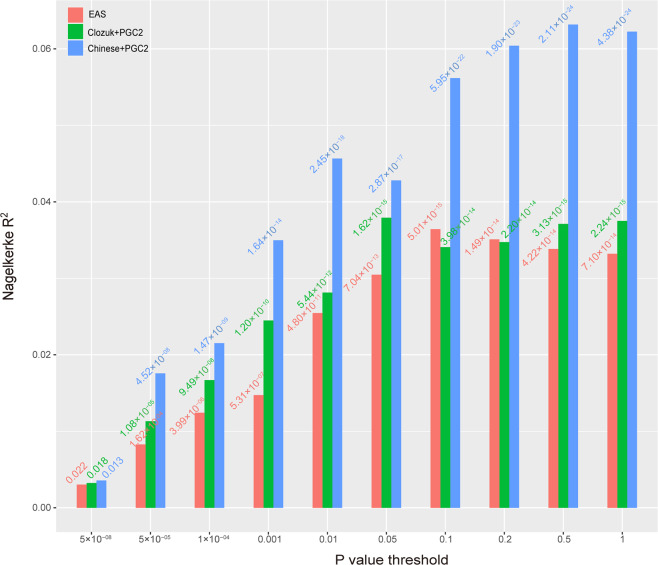
Genetic risk prediction accuracy in subgroup 1 EOS cases and controls (discovery stage) using different training sets. PRSs were computed using GWAS summary statistics from three training sets. The red plot represents PRS result from EAS training set (including 22,778 SCZ cases and 35,362 controls). The green plot represents PRS result from CLOZUK + PGC2 training set (including 40,675 SCZ cases and 64,643 controls). The blue plot represents PRS result from Chinese+PGC2 training set (including 43,175 SCZ cases and 65,166 controls).

All these training sets had good performance as the PRS scores derived from these datasets were significantly associated with the case–control status of our EOS samples in the discovery stage (Fig. 4 and Supplementary Fig. S12). The *P* values ranged from 0.018 to 2.24 × 10^−15^ when GWAS summary statistics from CLOZUK + PGC2 were used, ranged from 0.013 to 4.38 × 10^−24^ when GWAS summary statistics from Chinese+PGC2 were used, and the *P* values ranged from 0.022 to 7.10 × 10^−14^ when GWAS result from East Asian samples were used (Fig. 4). The genetic variance (estimated by Nagelkerke *R*^2^) ranged from 0.32% to 3.75% when CLOZUK+PGC2 training set was used, ranged from 0.36% to 6.23% when Chinese+PGC2 training set was used, and ranged from 0.30% to 3.32% when East Asian sample training set was used. Overall, the Chinese+PGC2 training set explained larger genetic variance (estimated by Nagelkerke *R*^2^ value) than CLOZUK+PGC2 and East Asian sample training sets. These results indicated that EOS and SCZ share substantial genetic basis and the GWAS results had significant power in associating the case–control status of our EOS samples. In addition, we also found that the training set containing Chinese SCZ samples had better performance power.

### Tissue and GO/KEGG gene set enrichment analysis by MAGMA

To explore if the GWAS associations of EOS are enriched in specific tissues or pathways, we utilized an online tool FUMA (https://fuma.ctglab.nl)^51^ to perform MAGMA^46^ tissue enrichment analysis in 53 GTEx tissues. Our results indicated that EOS GWAS associations were enriched in pituitary (*P* = 0.0048) and several other brain tissues such as brain cortex (*P* = 0.019) (Supplementary Fig. S13), suggesting that risk variants might contribute to EOS susceptibility by affecting genes expressed in these tissues.

We also performed that GO/KEGG enrichment analysis by using MAGMA gene set analysis. We found the EOS GWAS summary statistics are enriched in negative_regulation_of_axon_extension (*P* = 0.00013), negative_regulation_of_gliogenesis (*P* = 0.00028), regulation_of_mononuclear_cell_migration (*P* = 0.00092), negative_regulation_of_glial_cell_proliferation (*P* = 0.00069), and other GO terms (Supplementary Table S5), suggesting that the risk genes may confer risk of EOS by regulating these pathways.

## Discussion

In this study, we conducted the first GWAS of EOS in Chinese population and identified four risk loci for EOS. Among the four GWS loci, the functional variant rs1801133 showed the most significant association with EOS. Of note, previous studies have revealed that rs1801133 was associated with neurodegenerative and neuropsychiatric disorders, including late-onset Alzheimer’s disease^52^, Parkinson’s disease^53^, and depression^54^, suggesting the important role of rs1801133 in the risk of neurodegenerative and neuropsychiatric diseases. Interestingly, Vares et al.^55^ found that rs1801133 (C667T) was associated with the age of schizophrenia onset in a dose-dependent manner, with the T allele associated with lower age of onset. In our study, we also found that the A allele (corresponding to the T allele in study of Vares et al.) was the risk allele for EOS. In addition to genetic evidence, Roffman et al.^56^ found that rs1801133 disrupted prefrontal function in SCZ. These studies suggest that rs1801133 may represent an authentic risk variant for EOS.

We noticed that rs1801133 showed obvious allelic frequency differences between Northern Han (NH) and Southern Han (SH) Chinese in Chinese individuals included in the 1000 Genomes Project^35^. The frequencies of G allele in Chinese population are 53% (in NH) and 71% (in SH), respectively. In a recent study, rs1801133 also exhibited obvious frequency differences between NH and SH. The G allele frequencies in NH and SH were estimated to be 42.2% and 62.1%, respectively^57^. Thus, it is pivotal to control the population structure (or stratification) when investigating the associations between rs1801133 and diseases in Chinese population. In the discovery stage, we performed stringent PCA to select genetically matched cases and controls. The subjects included in our EOS discovery stage were mainly recruited from Henan province, which is located in the central part of China. However, in addition to recruiting controls from Henan province, we also recruited healthy controls from other provinces of China. Thus, a substantial number of controls were removed for subsequent GWAS (1366 controls were excluded while only 186 cases were excluded). Our final PCA analysis showed that there was no obvious population stratifications in our sample. Besides, we replicated the association between rs1801133 and EOS in an independent EOS sample. Of note, a previous GWAS study also showed a trend of association between rs1801133 and schizophrenia in Chinese population^18^. The fixed-effect *P* value between rs1801133 and schizophrenia was 2.36 × 10^−07^ (OR = 0.77, based on G allele) in the study by Yue et al.^18^, suggesting that this variant may be an authentic risk variant for SCZ. Furthermore, functional annotation analysis showed that rs1801133 may have regulatory function (Supplementary Table S6)^58^. However, more work is needed to validate our results. Replication in genetically independent populations (e.g., Europeans) and further functional characterization of rs1801133 will provide pivotal information about the role of rs1801133 in EOS.

In addition to rs1801133, rs592927 (which is located in the upstream of *RALGPS1*) also showed significant association with EOS. Interestingly, this SNP is associated with SCZ in European populations (*P* = 0.019, OR = 1.03, based on A allele)^9^, with the same risk allele as in our EOS. Of note, we noticed that a SNP (rs13284900) in LD (*r*^2^ = 0.77 in East Asians) with rs592927 showed GWS association with SCZ in a recent large-scale trans-ancestry meta-analysis (including East Asian and European samples) (*P* = 1.22 × 10^−09^, OR = 1.07, based on T allele)^9^. These evidences suggesting that this locus may be an authentic risk locus for SCZ in both Asian and European populations.

The four eQTL target genes of the GWS risk variants (*MTHFR, TDGF1, ANGPTL2*, and *RALGPS1*) play important roles in brain development and are associated with brain disorders. *MTHFR*-deficient mouse model study showed that both glutamate and γ-aminobutyric acid levels were reduced in mouse cerebellum and hippocampus region, indicating the important roles of *MTHFR* gene in regulating the level of neurotransmitters^59^. *TDGF1* gene encodes an epidermal growth factor-related protein, and loss-of-function mutation in *TDGF1* protein has been reported in human subjects with forebrain defects, indicating an important role of *TDGF1* in human brain development^60^. *ANGPTL2* encodes an angiopoietin-like protein and a recent study showed that *ANGPTL2* plays an important role in neuronal injury in acute ischemic brain^61^. The function of *RALGPS1* remains largely unknown. However, several studies showed that *RALGPS1* may play a role in brain disorders such as epileptic encephalopathy and intellectual disability^62,63^.

We performed sex-specific GWASs by splitting the samples into males and females (Supplementary Fig. S14). For male GWAS, there were 476 cases and 1,482 controls. For female GWAS, there were 780 cases and 1,179 controls. We performed a sex-specific association and identified 69 and 108 suggestive SNPs (*P* < 0.00001) in male and female GWASs, respectively. No overlapping SNP was found between the male and female suggestive SNPs. And only one SNP showed nominal association with females (*P* < 0.05) was identified in suggestive SNPs found in male GWAS. In addition, 14 SNP that showed nominal associations with EOS (*P* < 0.05) in males were found in the 108 suggestive SNPs in female GWAS. These results may reflect gender differences between male and female GWAS. However, due to the small sample size in the sex-specific study, further work are needed to explore this.

We replicated previously reported GWS variants (from a large-scale meta-analysis of East Asian and PGC European samples)^6^ in our discovery sample. We found that some previously reported GWS variants were also associated with EOS in our sample. Thus, our study provides further support for the involvement of these replicated risk variants in SCZ. However, it should be noted that the sample size of our study is relatively small. Therefore, only limited variants were replicated. Replication studies in larger sample are necessary and important. The full list of SNPs used for replication in our sample is provided in Supplementary Table S7.

Our PRS analysis showed that the GWAS summary statistics from previous SCZ GWASs had good performance as the PRS score were significantly associated with the status of our EOS case and healthy controls. These results indicated that the late or adulthood-onset SCZ and EOS shared substantial genetic basis. Of note, the Chinese+PGC2 training set had better performance than CLOZUK+PGC2 and EAS datasets in power, which was likely due to that the Chinese+PGC2 training set included substantial Chinese ancestry samples, and our EOS samples were all Chinese ancestry. In addition, compared with EAS, Chinese+PGC2 had a larger sample size (which may improve the performance of PRS analysis). By contrast, CLOZUK+PGC2 dataset was primarily derived from populations of European ancestry. We also noticed that the proportion of variance explained decreased when we converted the Nagelkerke *R*^2^ into liability scale^64^ (Supplementary Fig. S15).

There are several limitations in this study. First, the sample size of this study is still relatively small compared with other GWASs on psychiatric disorders. Considering that only about 5% SCZ cases have an illness onset before age 18, it is quite difficult and challenge to recruit adequate EOS cases to conduct GWASs. Replication of our findings in large-scale GWASs with more EOS cases will provide further evidence for the involvement of the identified risk loci in EOS. Second, we did not conduct a full meta-analysis using the samples from the discovery and replication stages. A major reason for this is that different genotyping platforms were used to genotype the cases (ASA) and controls (GSA) in our replication stage. To avoid the potential batch effect of different genotyping platforms, we did not perform GWAS in the replication samples. In addition, we noticed that the number of overlapping SNPs from ASA and GSA platforms were relatively small (N = 133,620). Although imputation may solve this problem, currently the cases and controls of most GWASs were genotyped with the same genotyping platform. It is inappropriate to perform a GWAS using cases and controls genotyped with different platforms. Further efforts are warranted to perform genotyping using the same platforms, which will facilitate a full meta-analysis in larger samples and includes more genetic variants. Finally, by using eQTL analysis, we identified several potential target genes (including *MTHFR, TDGF1, ANGPTL2*, and *RALGPS1*) of the identified risk variants. Nevertheless, currently we do not know how the risk variants confer risk of SCZ. More work is needed to elucidate the roles and mechanisms of these risk variants in SCZ.

In summary, we conducted a GWAS of EOS and identified four risk loci. Our study identified novel risk variants for EOS and our findings showed that EOS and SCZ shared substantial genetic basis. Our study provides new insights for the genetic basis of EOS. Replication of the results of our findings in independent populations and functional characterizations will help explore new targets for therapeutics and diagnostics of EOS.

## Supplementary information

Supplementary Materials
