# Supplementary material for: Genome wide association study identifies four loci for early onset schizophrenia
Source: Transl Psychiatry. 2021 Apr 27;11:248. doi: 10.1038/s41398-021-01360-4 (PMC8079394; doi:10.1038/s41398-021-01360-4)
Supplement: Supplementary file 1 — Supplementary Materials [file 41398_2021_1360_MOESM1_ESM.doc]

**Supplementary Materials for**

**Genome wide association study identifies four loci for early onset schizophrenia**

Suqin Guo^1,2^, Jiewei Liu^3^, Wenqiang Li^1,2^, Yongfeng Yang^1,2^, Luxian Lv^1,2^, Xiao Xiao^3^, Ming Li^3^, Fanglin Guan^4^ , Xiong-Jian Luo^3,5,6^

**Supplementary Contents:**

**1: Supplementary Methods**

**2: Supplementary Figures**

**Figure S1.** The flow chart of the quality control processes of this study.

**Figure S2.** PCA of our GWAS samples and samples from the 1000 Genomes project.

**Figure S3.** PCA of all EOS cases and healthy controls included in the discovery stage.

**Figure S4.** PCA results of subgroup 1 (N=860 EOS cases and 1,505 controls).

**Figure S5.** PCA results of subgroup 2 (N=396 EOS cases and 1,156 controls).

**Figure S6.** PCA result of our subgroup1 samples with 1000 genome CHB and CHS samples. **Figure S7.** PCA result of our subgroup2 samples with 1000 genome CHB and CHS samples. **Figure S8.** QQ plot of subgroup 1 in discovery stage (N=860 EOS cases and 1,505 controls). **Figure S9.** QQ plot of subgroup 2 in discovery stage (N=396 EOS cases and 1,156 controls). **Figure S10.** QQ plot of subgroup 1 and subgroup 2 (N=1,256 EOS cases and 2,661 healthy controls).

**Figure S11.** Boxplot of eQTL analysis.

**Figure S12.** PRS analysis of the subgroup2 in the discovery stage.

**Figure S13.** MAGMA tissue enrichment analysis result of EOS GWAS summary statistics based on 53 GTEx tissues

**Figure S14.** Gender differences between male and female EOS for the GWAS results.

**Figure S15.** PRS analysis of the subgroup1 in the discovery stage.

**3: Supplementary Tables**

**Table S1.** The sample information of cases and controls in discovery and replication stages.

**Table S2.**The SNPs that showed suggestive association (*P*<1.0×10^-4^) with EOS in two-stage analysis (discovery and replication).

**Table S3.** Expression quantitative trait loci (eQTL) analysis of GWS risk variants.

**Table S4.** eQTL and meQTL of *MTHFR* that show association with EOS.

**Table S5.** MAGMA GO/KEGG enrichment analysis of EOS GWAS summary statistics (top10 is shown).

**Table S6.** Functional annotation of the index snps of four EOS GWS loci by RegulomeDB.

**Table S7.** The association significance of genome wide significant risk variants (from a recent large-scale trans-ancestry meta-analysis of East Asians and PGC2 Europeans) with EOS in our discovery stage.

1. **Supplementary Methods**

***DNA extraction and genotyping***

Genomic DNA was extracted from peripheral blood with the standard phenol-chloroform method. All of the EOS cases and controls in the discovery stage were genotyped with the Illumina Genome-Wide Asian Screening Array (ASA-MD) Chips by Beijing Guoke Biotechnology Co., LTD ([www.bioguoke.com](http://www.bioguoke.com/)). The EOS cases of the replication stage were genotyped with ASA Chips, while the controls were genotyped with the Illumina Infinium Global Screening Array (GSA) SNP array platforms (Beijing Guoke Biotechnology Co., LTD). The DNA samples were incubated with probes on Illumina SNP chip and the SNP chip was then scanned by Illumina iScan systems, this toolkit outputs probe intensities file in idat format. And the idat files were further processed using the GenomeStudio software to perform data visualization and genotype data output.

***Brain eQTL datasets and PsychEncode dataset***

The first brain eQTL dataset was from the Common Mind Consortium (CMC)^1^. Briefly, brain issues (DLPFC) of 592 individuals were collected by CMC and gene expression was quantified with RNA-seq, gene expression level was normalized by CPM (read counts per million total reads). Genotyping was performed by Illumina Infinium HumanOmniExpressExome 8 v 1.1b and eQTL analysis (from 467 European-ancestry subjects) was performed by MatrixEQTL^2^. The second brain eQTL dataset was from study of Jaffe *et al*^3^ (referred as LIBD dataset). Briefly, this eQTL data was from eQTL analysis of 412 subjects (dorsolateral prefrontal cortex). Gene expression levels were measured by RPKM (Reads Per Kilobase Per Million mapped reads). Genotyping was implemented by three platforms, including HumanHap650Y_V3, Human 1M-Duo_V3, and Omni5 BeadChips. And eQTL analysis was conducted by using R package MatrixEQTL and only 412 (age >13) samples were included^2^. More detailed information about how the sample collection and data processing steps, please refer to the descriptions in the original papers^1, 3^.

PsychEncode collected human brain tissues of multiple subjects, including individuals with psychiatric disorder and normal subjects. Gene expression level in cortical tissues were quantified with RNA sequencing. Expression data from 559 SCZ cases and 936 controls were used for differential expression analysis. More detailed information about PsychEncode (including collection of subjects and brain tissues, RNA sequencing, data processing and statistical analyses) can be found in the original papers^4^ and on the PsychEncode website: <http://www.psychencode.org/>.

In addition, we also examined if SNPs associated with *MTHFR* expression (CMC dataset)^1^ or methylation (quantitative trait loci, meQTLs)^5^ *(*from Hannon *et al*) are also associated with EOS in our EOS GWAS.

***Polygenic risk scoring (PRS) analysis***

GWAS results from three studies were used as the training sets. The first training dataset was the summary statistics from the study of Pardinas *et al* ^6^ (referred as CLOZUK + PGC2). This study conducted a meta-analysis through combining the genome-wide association results from the CLOZUK samples (11,260 cases and 24,542 controls) and PGC2 dataset (35,476 cases and 46,839 controls). Pardinas *et al* identified 50 novel genetic risk loci for SCZ. The second training set was from a recent study by Li *et al*^7^ (referred as Chinese+PGC2). Li *et al*. performed a trans-ancestry GWAS meta-analysis through combining Chinese samples (7,699 cases and 18,327 controls) and PGC2 samples (35,476 cases and 46,832 controls) and identified 30 novel genome-wide significant loci for SCZ. The third training set was from a recent GWAS study of East Asian ancestry samples (referred as EAS)^8^, this study collected 22,778 SCZ cases and 35,362 controls in total and 21 genome-wide-significant sites were identified. The target dataset is the genotype data of EOS cases and controls included in the discovery stage. Strand ambiguous SNPs (A/T, G/C) were removed when perform PRS analysis. We set 10 *P*-value thresholds, including 5.00×10^-08^, 5.00×10^-05^, 1.00×10^-04^, 0.001, 0.01, 0.05, 0.1, 0.2, 0.5 and 1. The variance was estimated by Nagelkerke R^2^ and *P* values were estimated by permutation test. The clumping parameters of PRSice 2.0 used in this study were as follows: --clump-kb 250, --clump-p 1.0, --clump-r2 0.10. The Nagelkerke’s R^2^ is based on the observed scale. We converted Nagelkerke’s R^2^ into liability scale^9^, assuming that the population risk is 0.0005 for EOS.

***LD score regression analysis***

LDSC can be used to distinguish confounding from polygenicity in GWASs^10^. Low intercept indicates that the observed genomic inflation is mainly attributable to polygenicity rather than confounding^10^. The summary statistics from subgroup 1 were used and the reference LD scores of East Asian ancestry were downloaded from LDSC website (<https://data.broadinstitute.org/alkesgroup/LDSCORE/>). The population risk was set to 0.0005 (suppose that the prevalence of SCZ is 1%, and 5% SCZ cases are EOS).

***Tissue and GO/KEGG enrichment analysis by MAGMA***

To explore if the EOS GWAS associations are enriched in specific tissues or pathways, we performed tissue and GO/KEGG enrichment analysis by MAGMA software^11^.

For tissue enrichment analysis, we utilized an online tool FUMA software (<https://fuma.ctglab.nl/>)^12^. MAGMA (implemented FUMA) uses expression data from 53 GTEx tissues for enrichment analysis. More detailed about the tissue specific gene set calculation, module usage, please refer to the original FUMA paper and the website (<https://fuma.ctglab.nl/>).

As no KEGG items were included in FUMA-MAGMA analysis module, we download GO and KEGG items from MSigDB database (v7.1) (<http://software.broadinstitute.org/gsea/msigdb/>), which includes 10,378 gene sets. The gene sets with the number of genes ranging from 10 to 200 were remained for further analysis. MAGMA software were downloaded from MAGMA website (https://ctg.cncr.nl/software/magma)^11^.

**2: Supplementary Figures**


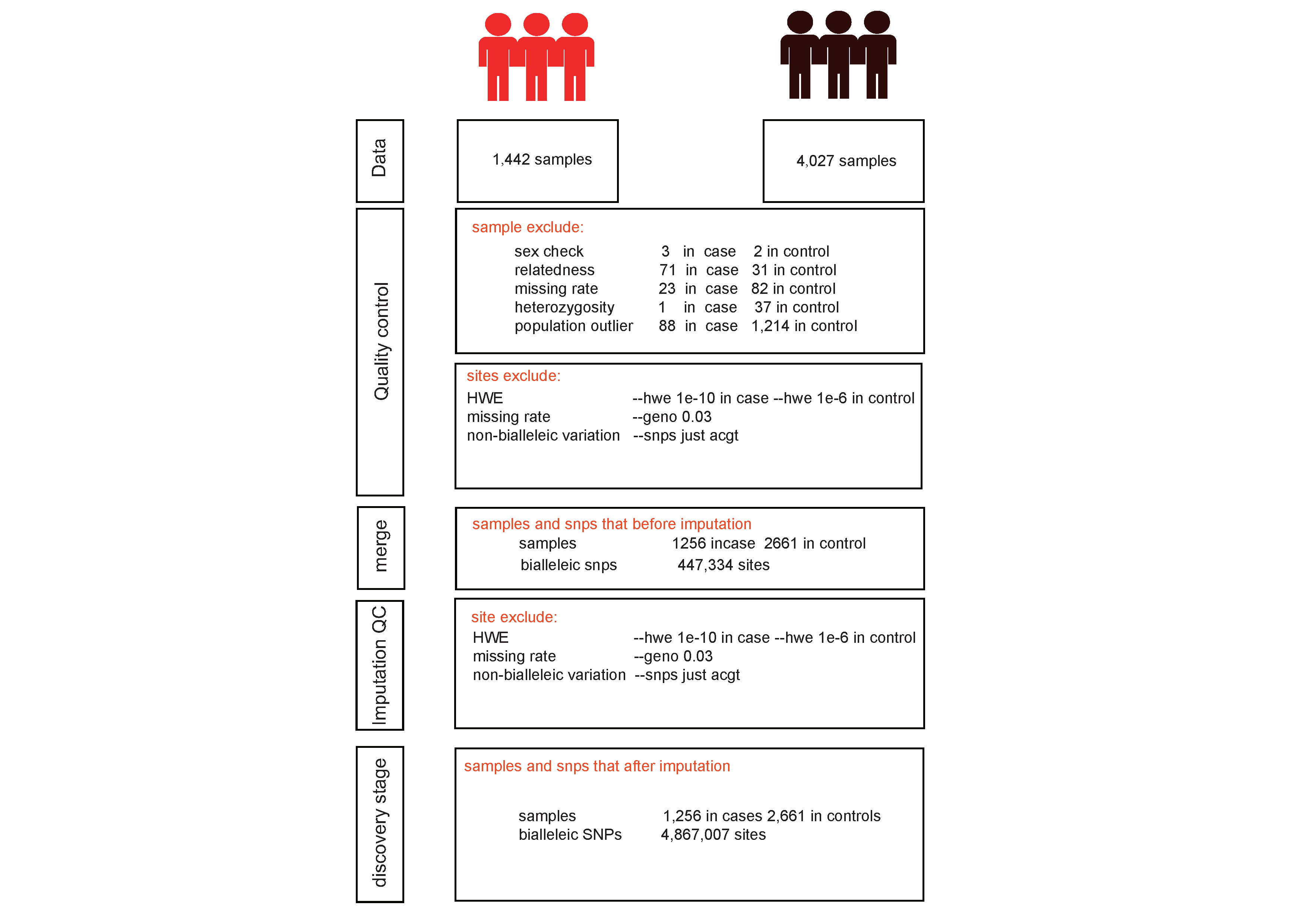


**Figure S1. The flow chart of the quality control processes of our samples**


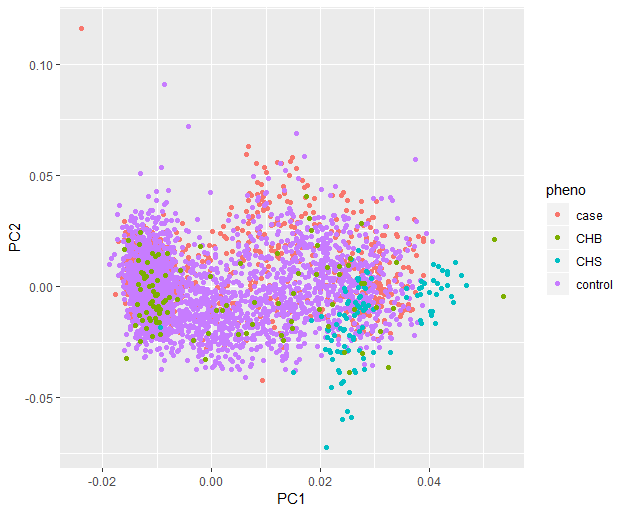

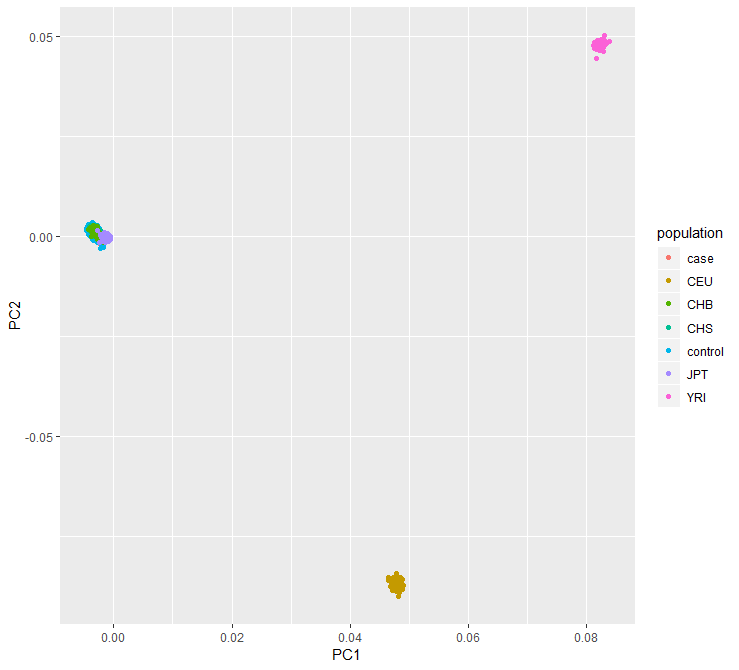


**Figure S2. PCA of our samples and samples from the 1000 Genomes project.** The samples included in the discovery stage and samples from the east Asian populations (including CHB, CHS and JPT) formed a dense cluster, indicating that the samples included in the discovery stage were of Han Chinese ancestry. We zoom in the case and control samples pca map by performed the pca of our samples with 1000 genome CHB and CHS samples.


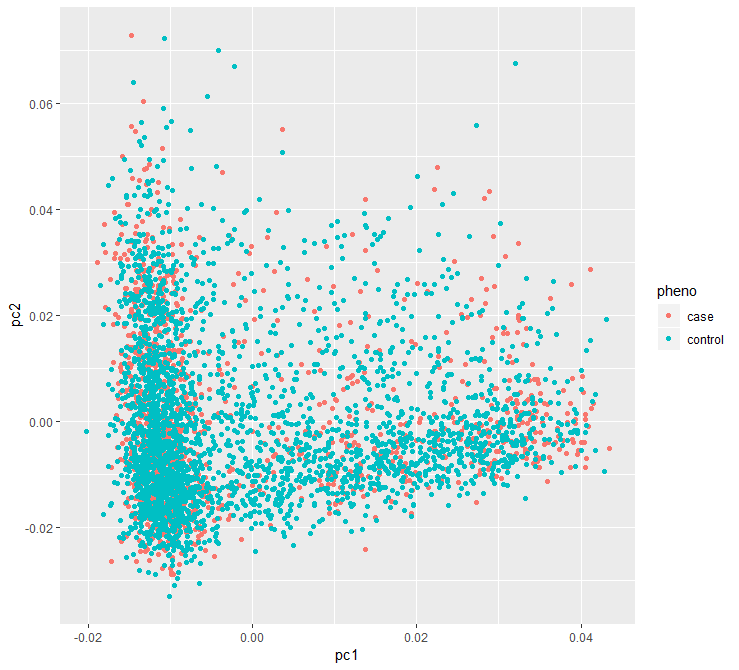


**Figure S3. PCA of all EOS cases and healthy controls included in the discovery stage.** PCA analysis identified two clusters (subgroups) in our EOS cases and controls (suggesting subtle population stratification), we thus divided our discovery sample into two genetically matched subgroups and performed GWAS separately.


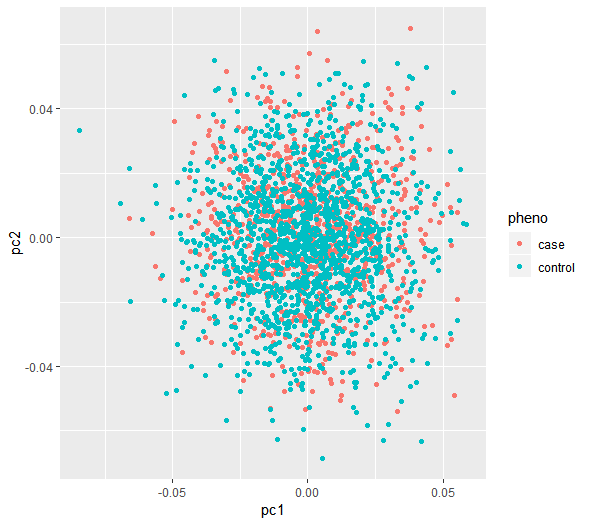


**Figure S4. PCA results of subgroup 1 (N=860 EOS cases and 1,505).** PCA analysis showed that there was no stratification in subgroup 1.


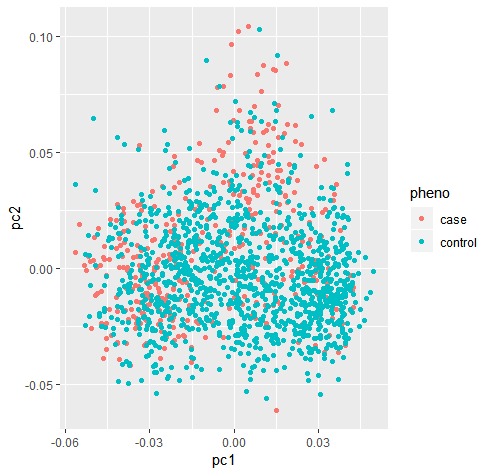


**Figure S5. PCA results of subgroup 2 (N=396 EOS cases and 1,156 controls).** PCA analysis showed that there was no stratification in subgroup 2.


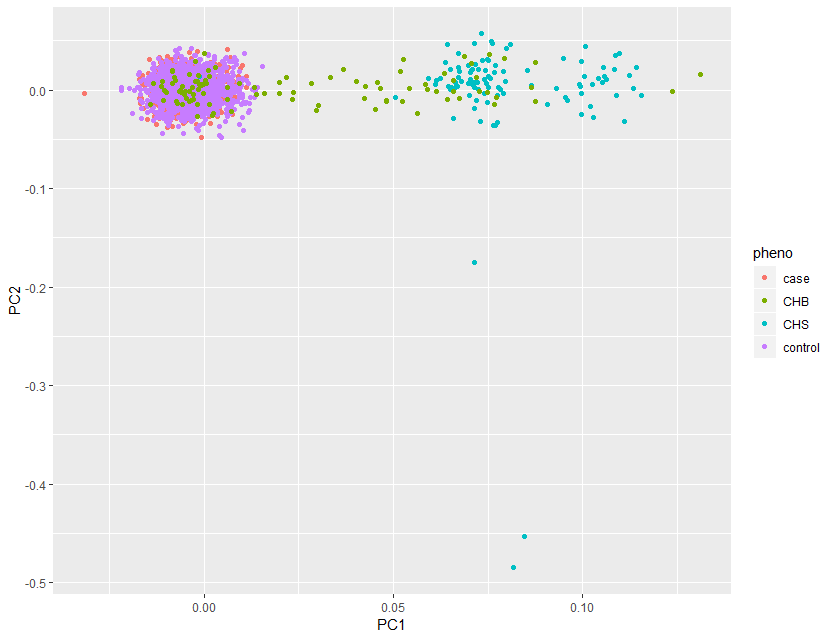


**Figure S6. PCA result of our subgroup1 samples with 1000 genome CHB and CHS samples.**


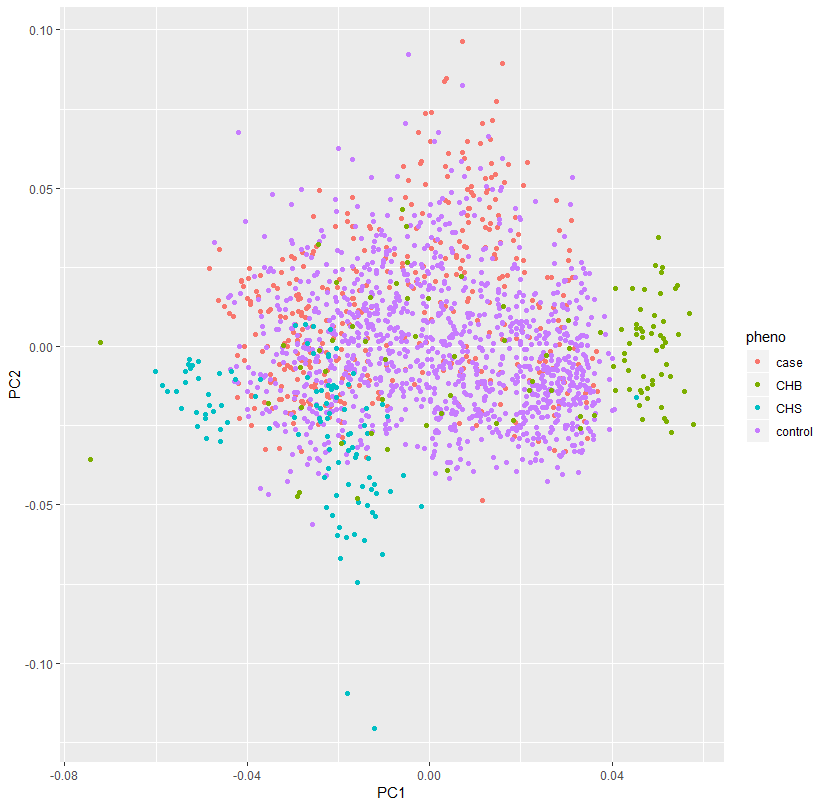


**Figure S7. PCA result of our subgroup2 samples with 1000 genome CHB and CHS samples.**


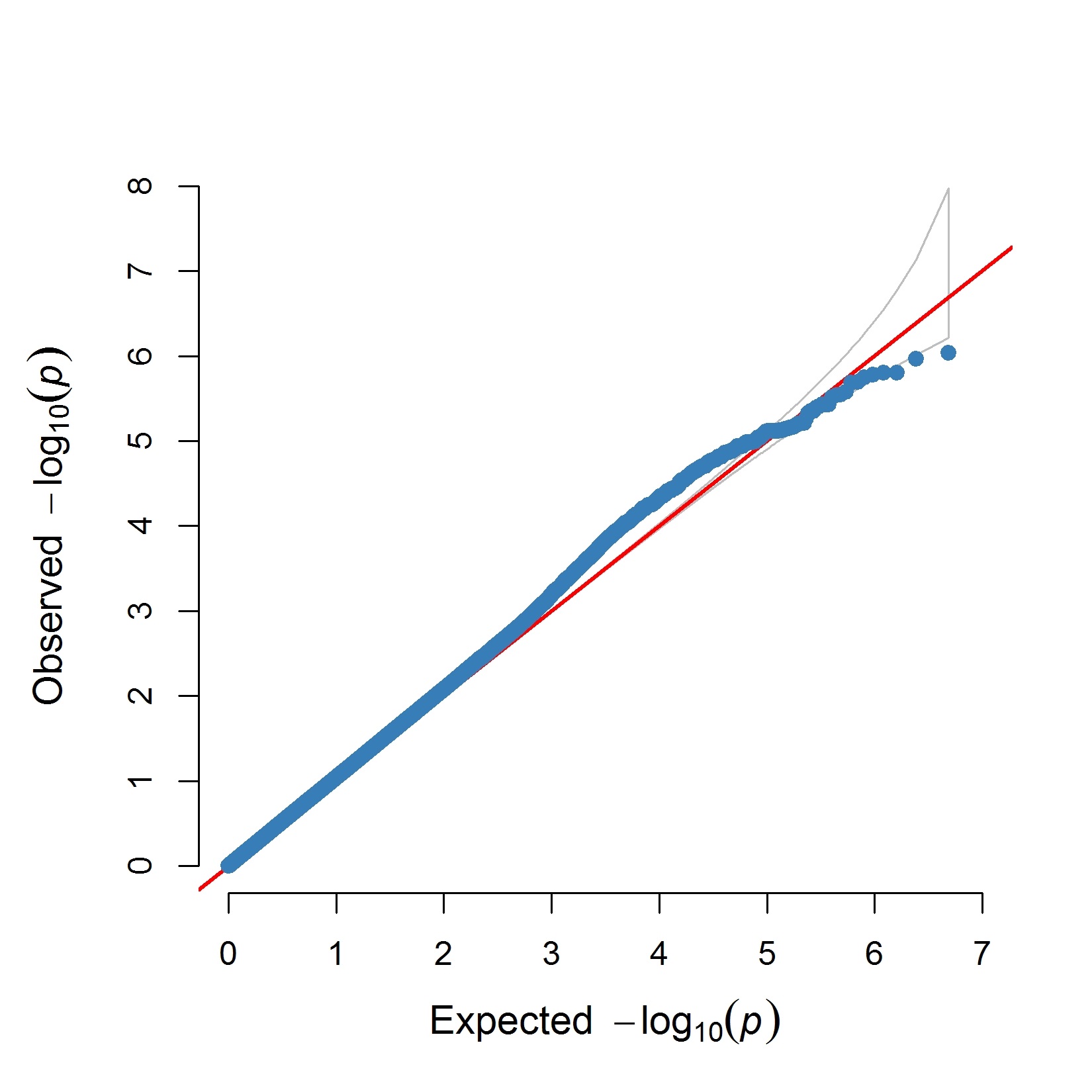


**Figure S8. QQ plot of subgroup 1 in discovery stage (N=860 EOS cases and 1,505 controls).**


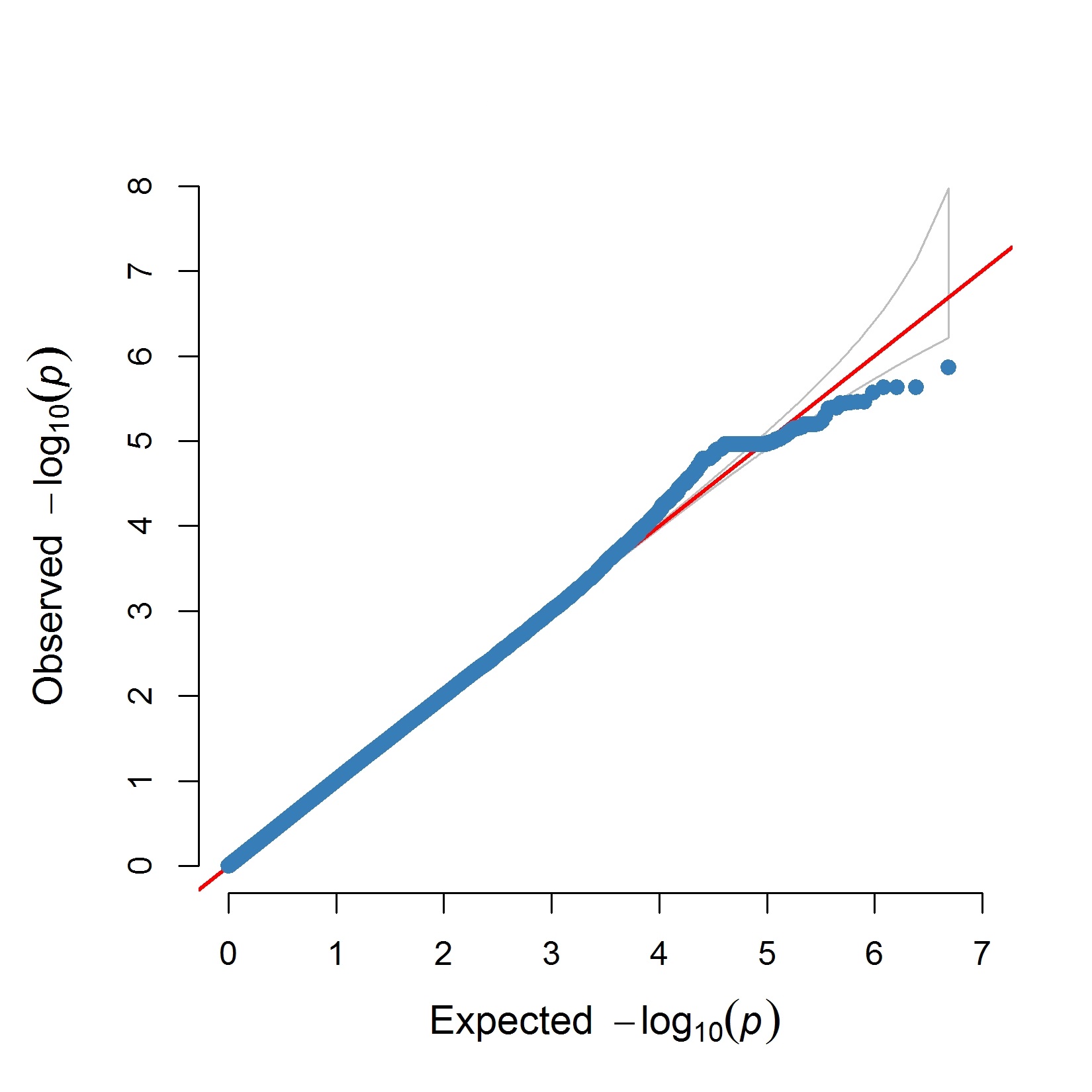


**Figure S9. QQ plot of group 2 in discovery stage (N=396 EOS cases and 1,156 controls).**


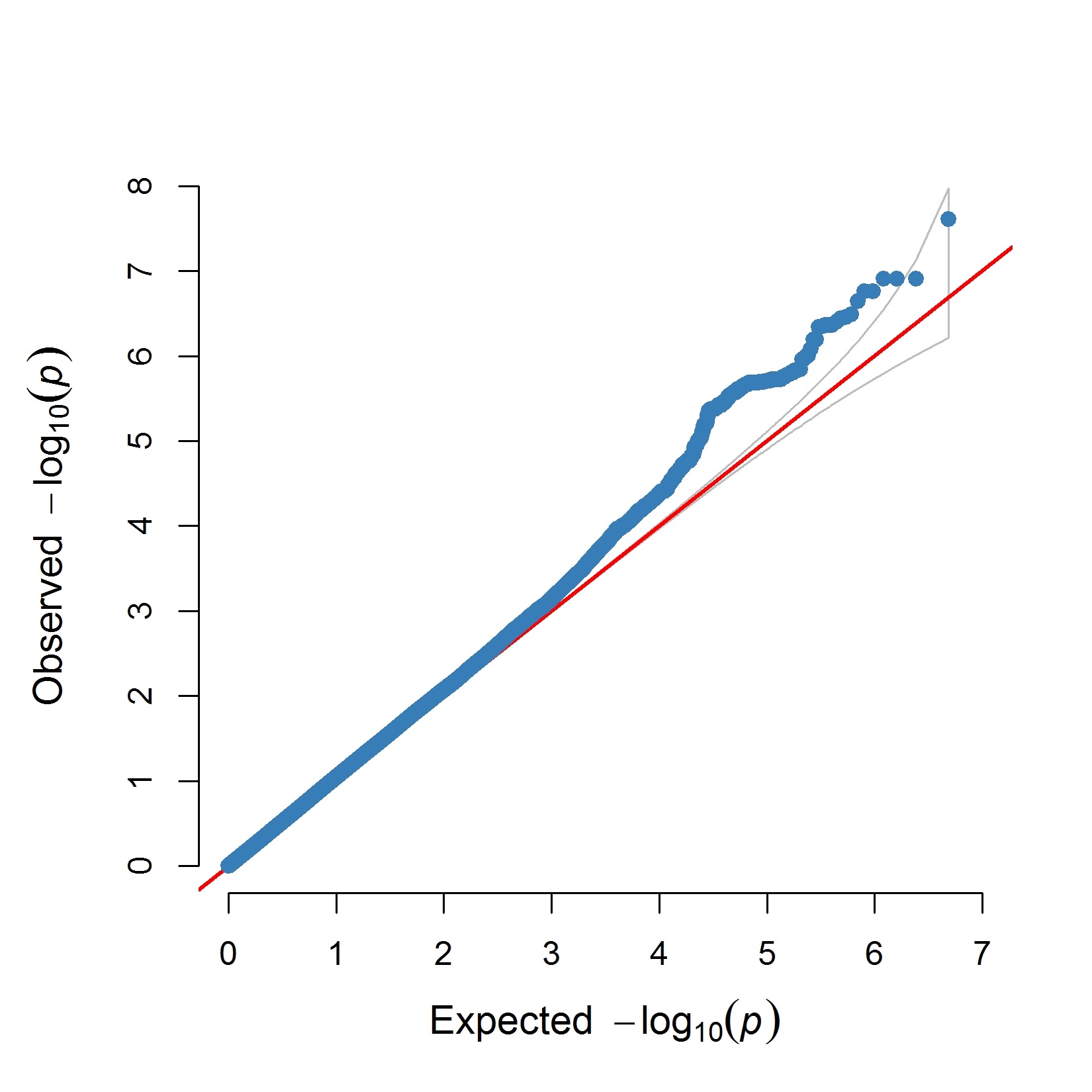


**Figure S10. QQ plot of subgroup 1 and subgroup 2 (N=1,256 EOS cases and 2,661 healthy controls).**


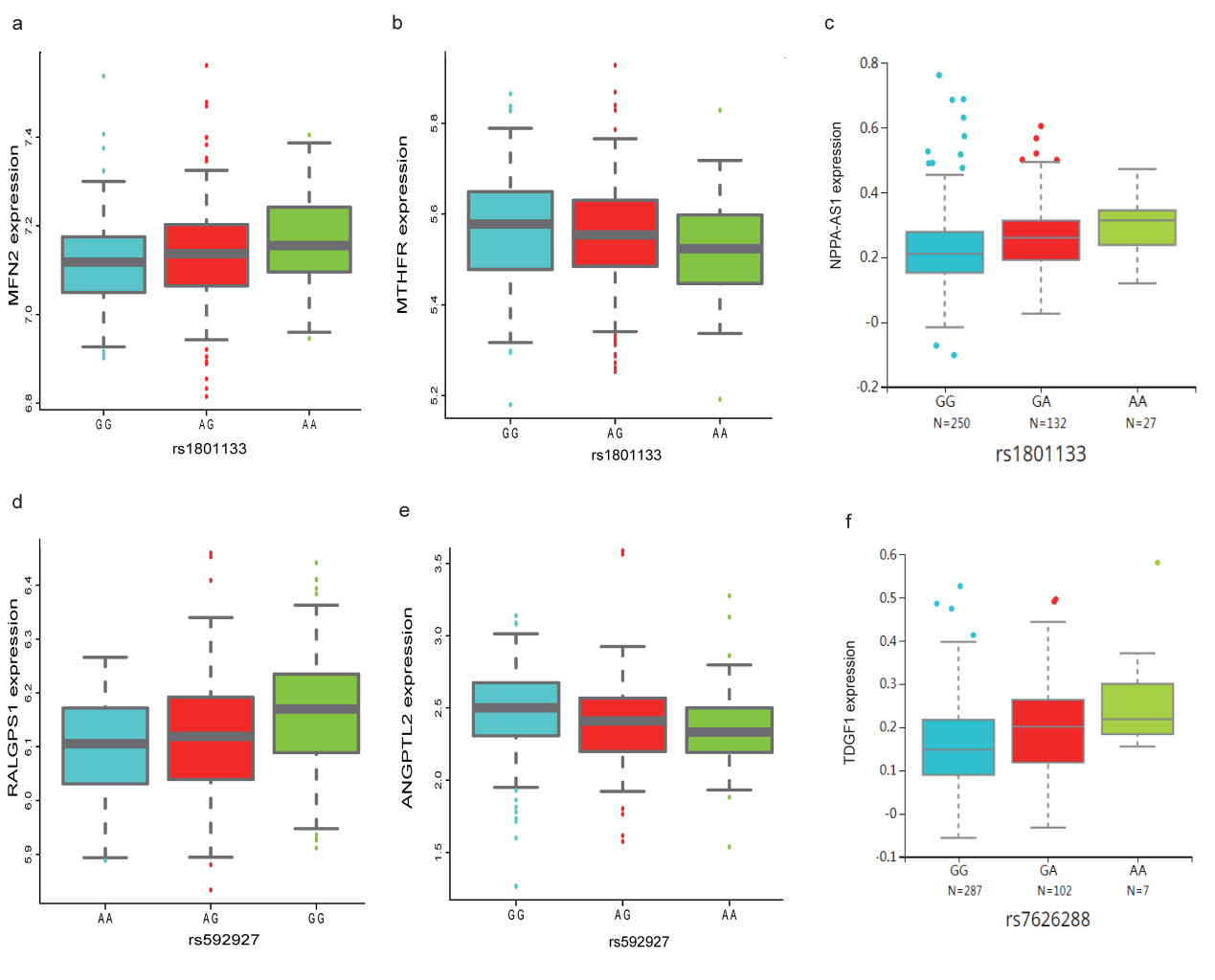


**Figure S11. Boxplot of eQTL analysis.**


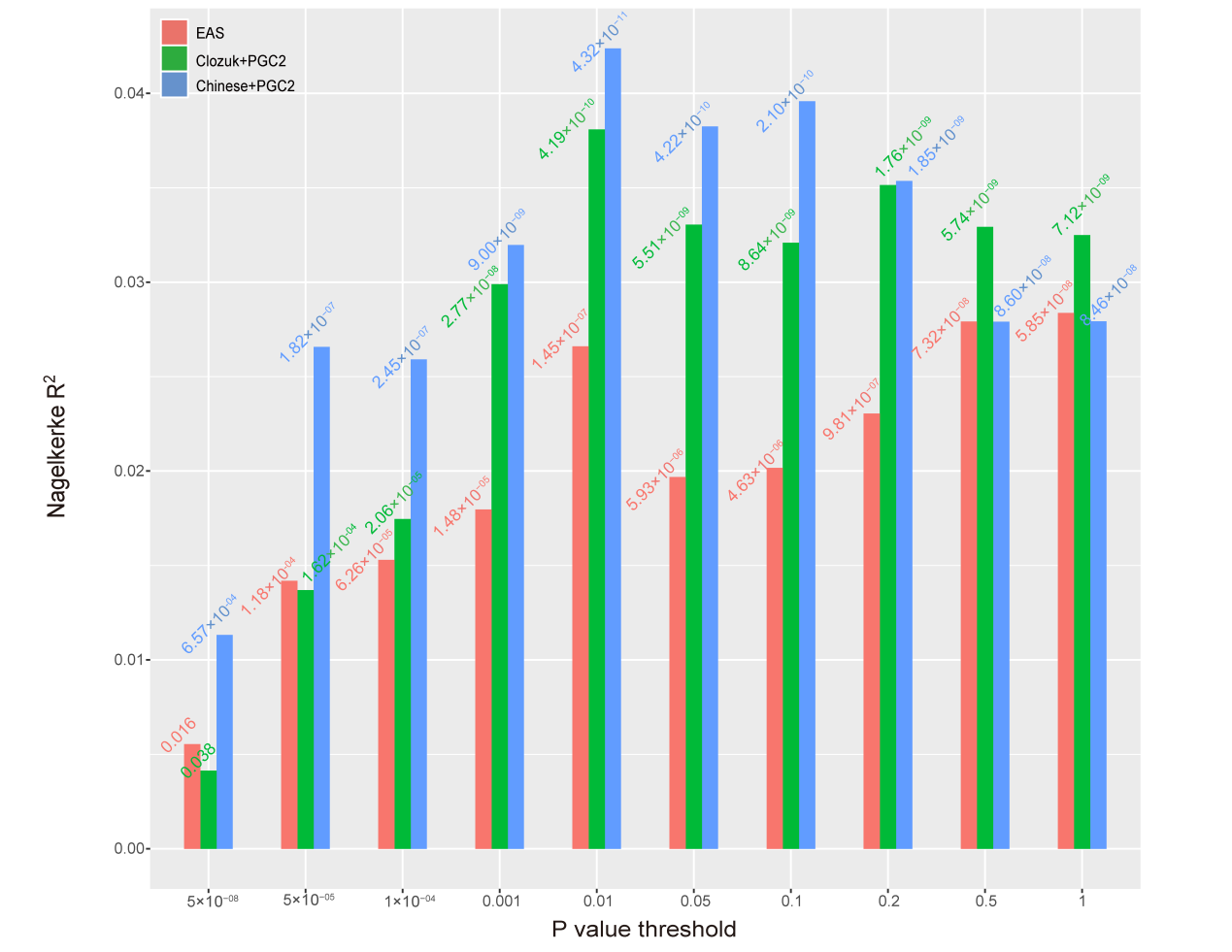


**Figure S12. PRS analysis of the subgroup2 in the discovery stage.**


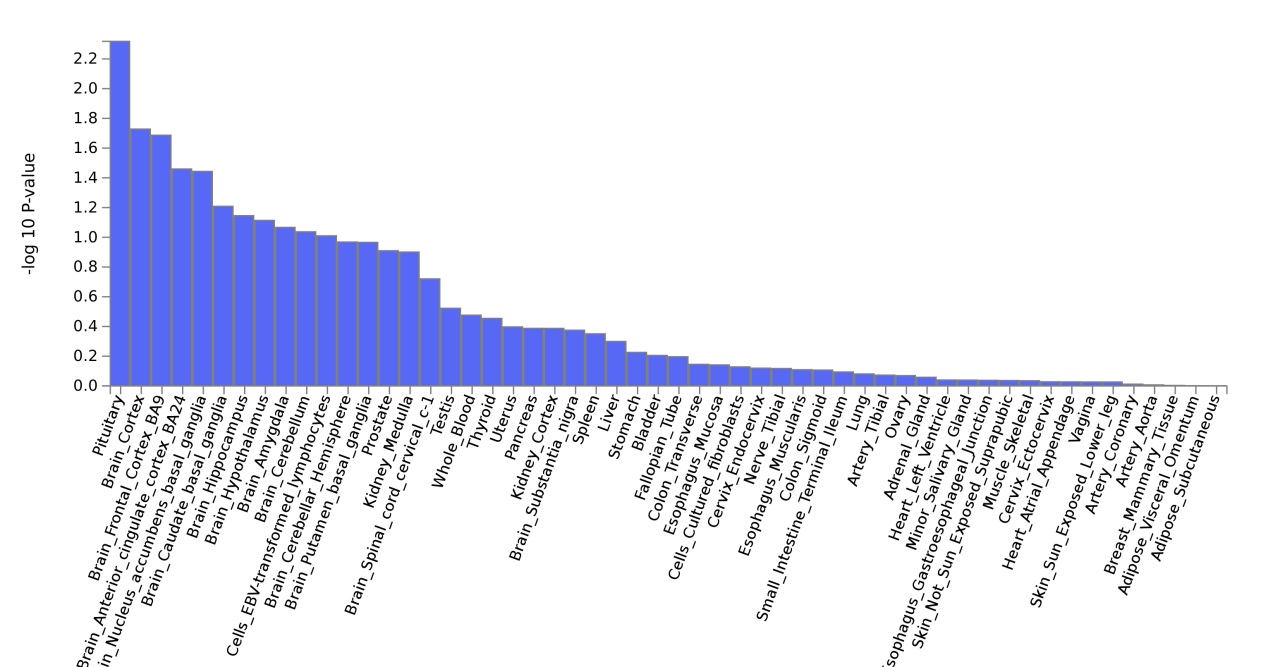


**Figure S13. MAGMA tissue enrichment analysis result of EOS GWAS summary statistics based on 53 GTEx tissues**


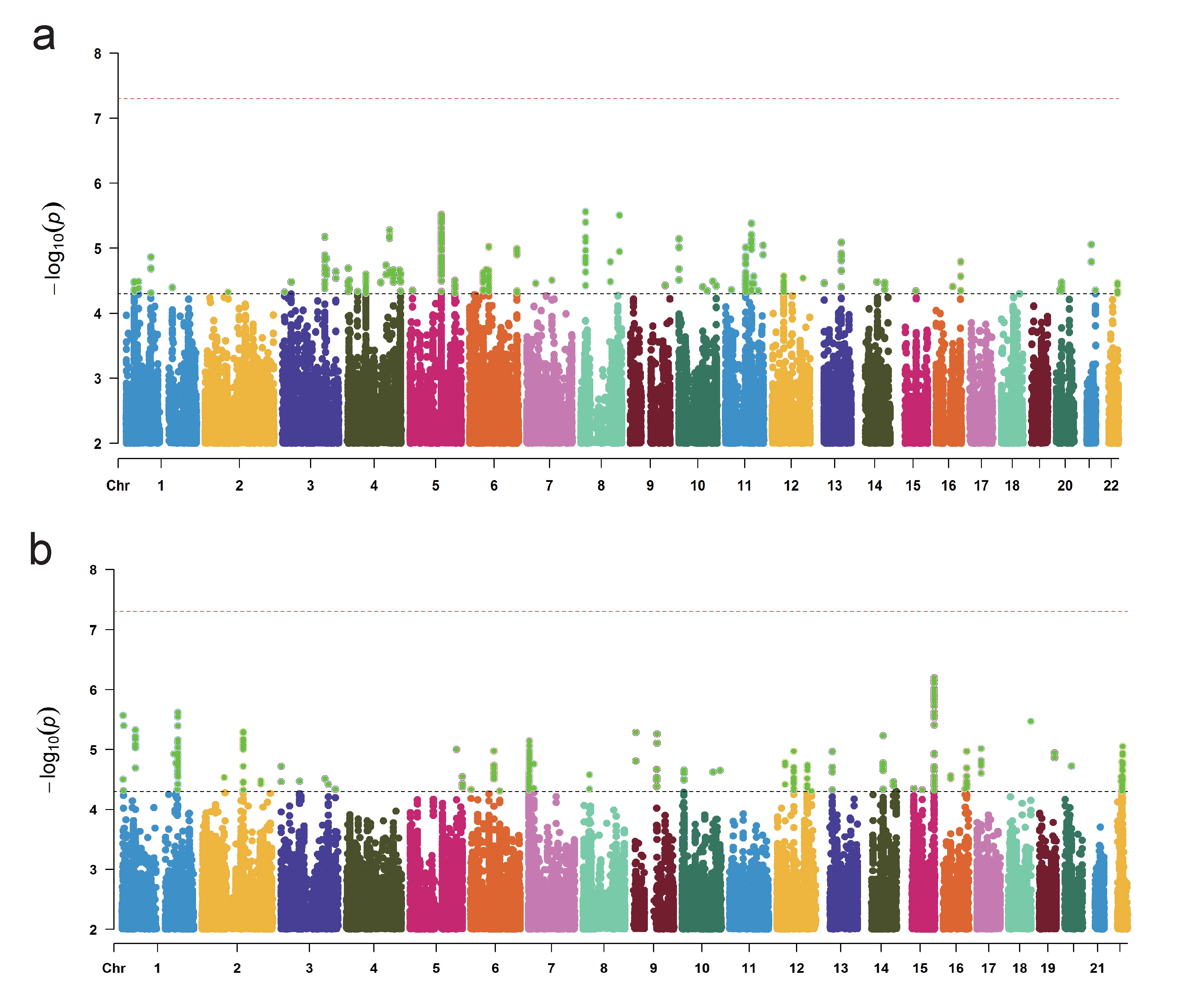


**Figure S14. Gender differences between male and female EOS for the GWAS results**. a, Manhattan plot of male EOS GWAS result; b, Manhattan plot of female EOS GWAS result;


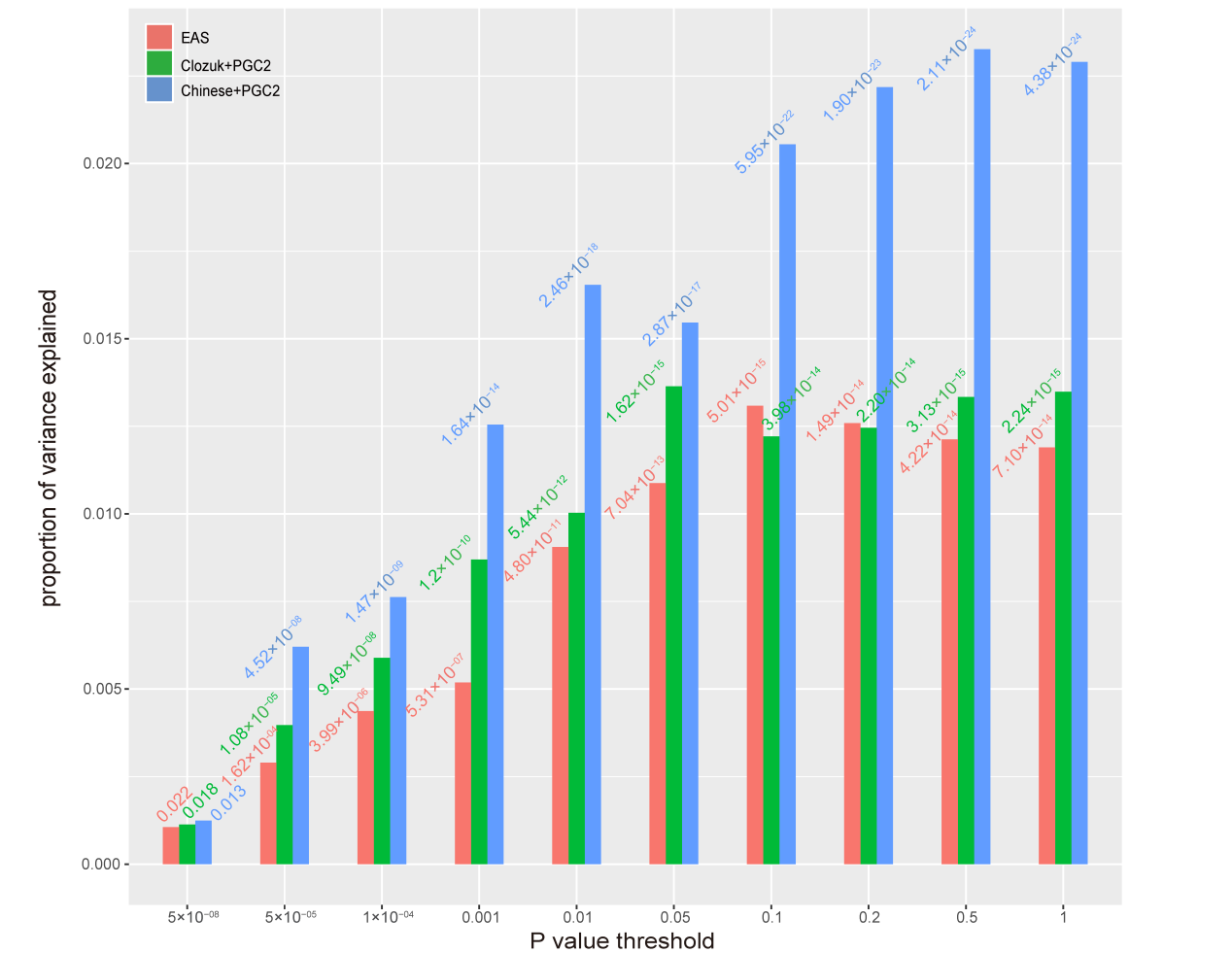


**Figure S15. PRS analysis of the subgroup1 in the discovery stage.** The y-axis is the proportion of variance explained based on the liability scale (assuming the population risk of EOS is 0.0005 ).

**3: Supplementary tables**

**Table S1. The sample information of cases and controls in discovery and replication stages**

| **Sample status** | **Analysis stages** | **Sample size** | **Mean age*(s.d.)** | **Male/Female** |
| --- | --- | --- | --- | --- |
| EOS cases | Discovery stage | 1,256 | 14.57 (2.27) | 41%/59% |
|  | Replication stage | 903 | 14.39 (1.94) | 36%/64% |
| Controls | Discovery stage | 2,661 | 28.60 (7.01) | 55%/45% |
|  | Replication stage | 3,900 | 28.55 (6.51) | 54%/46% |

*For cases, it indicates the onset age of EOS patients

**Table S2. The snps that show suggestive association (P<1.0×10^-4^**) with EOS in two stages analysis (discovery and replication)

| **chr** | **position(hg19)** | **SNP** | **A1** | **A2** | **p-value** | **OR** |
| --- | --- | --- | --- | --- | --- | --- |
| 1 | 11856378 | rs1801133 | A | G | 4.03E-15 | 1.346 |
| 1 | 82915881 | rs2791670 | A | G | 7.99E-08 | 1.2218 |
| 1 | 82919925 | rs1281571 | A | G | 4.14E-08 | 1.2271 |
| 1 | 82923142 | rs58579550 | A | G | 5.90E-08 | 1.2242 |
| 1 | 82934127 | rs34756544 | C | G | 1.31E-05 | 1.1908 |
| 1 | 82934252 | rs3908529 | G | A | 1.24E-05 | 1.1914 |
| 1 | 82934401 | rs3863840 | G | A | 1.33E-05 | 1.1907 |
| 1 | 82934675 | rs67896212 | A | G | 1.60E-05 | 1.1885 |
| 1 | 82934717 | rs68129997 | G | A | 1.60E-05 | 1.1885 |
| 1 | 82935354 | rs35491247 | C | T | 1.60E-05 | 1.1885 |
| 1 | 82935780 | rs36106802 | A | G | 1.60E-05 | 1.1885 |
| 1 | 82939780 | rs3863841 | A | G | 4.46E-05 | 1.1774 |
| 2 | 200813584 | rs281773 | C | T | 9.33E-05 | 0.8514 |
| 2 | 200818928 | rs67647314 | A | G | 8.56E-05 | 0.8517 |
| 2 | 200819812 | rs281765 | C | T | 6.21E-05 | 0.849 |
| 3 | 46485369 | rs7626288 | A | G | 1.57E-09 | 0.7962 |
| 3 | 46498975 | rs17078864 | T | C | 1.28E-08 | 0.8007 |
| 3 | 64107627 | rs704386 | C | T | 7.70E-06 | 1.2264 |
| 3 | 64116041 | rs161660 | G | C | 7.03E-06 | 1.2303 |
| 3 | 70170588 | rs59260955 | G | A | 3.80E-05 | 1.1751 |
| 3 | 70172654 | rs9817459 | G | C | 3.75E-05 | 1.1752 |
| 3 | 70175431 | rs13327169 | G | A | 4.20E-05 | 1.1741 |
| 3 | 70176819 | rs9868919 | A | G | 5.22E-05 | 1.1718 |
| 3 | 70178988 | rs7340562 | A | G | 5.48E-05 | 1.1714 |
| 3 | 70182149 | rs9824741 | A | G | 5.35E-05 | 1.1716 |
| 3 | 70182179 | rs9880622 | C | T | 5.35E-05 | 1.1716 |
| 3 | 72535823 | rs7628911 | T | C | 3.41E-05 | 1.2898 |
| 4 | 80190131 | 4:80190131 | T | G | 1.10E-07 | 0.7927 |
| 4 | 80191417 | rs35840311 | G | A | 1.07E-07 | 0.7927 |
| 4 | 80192824 | rs1484141 | C | T | 9.48E-08 | 0.791 |
| 4 | 80193148 | rs28409854 | G | A | 3.89E-07 | 0.7714 |
| 4 | 80193152 | rs28711203 | G | A | 4.22E-07 | 0.7724 |
| 4 | 80201409 | 4:80201409 | A | T | 3.96E-07 | 0.7989 |
| 4 | 80201410 | 4:80201410 | A | T | 3.96E-07 | 0.7989 |
| 4 | 80201411 | 4:80201411 | A | T | 3.96E-07 | 0.7989 |
| 4 | 80201412 | 4:80201412 | A | T | 3.96E-07 | 0.7989 |
| 4 | 80201413 | 4:80201413 | A | T | 3.54E-07 | 0.7981 |
| 4 | 80204953 | rs1542636 | T | A | 1.11E-07 | 0.7908 |
| 4 | 161825002 | rs4295221 | C | T | 8.31E-05 | 1.1761 |
| 5 | 123452759 | rs10038458 | A | G | 7.61E-05 | 0.8554 |
| 5 | 123459983 | rs930889 | T | C | 8.75E-06 | 0.8384 |
| 5 | 157466598 | rs1957565 | T | C | 1.87E-05 | 1.2632 |
| 5 | 157467325 | rs3117730 | G | A | 1.87E-05 | 1.2632 |
| 5 | 157467717 | rs3117729 | T | C | 3.60E-05 | 1.209 |
| 5 | 157468107 | rs3096012 | C | T | 1.87E-05 | 1.2632 |
| 5 | 157468185 | rs3117728 | C | T | 1.87E-05 | 1.2632 |
| 5 | 157472345 | rs1040924 | G | A | 2.80E-05 | 1.2123 |
| 5 | 157474368 | rs1957564 | T | C | 2.80E-05 | 1.2123 |
| 5 | 157487568 | rs1002566 | C | T | 6.75E-05 | 1.201 |
| 5 | 157489768 | rs2419387 | T | C | 8.33E-05 | 1.1983 |
| 7 | 18683672 | rs3801983 | C | T | 2.19E-05 | 1.1694 |
| 7 | 18739502 | rs4719538 | A | C | 8.14E-05 | 1.1845 |
| 8 | 27717160 | rs12546118 | C | T | 1.04E-05 | 1.2815 |
| 8 | 27718347 | rs17058030 | T | C | 5.95E-05 | 1.2741 |
| 8 | 27720284 | rs73559379 | C | T | 8.81E-05 | 1.2671 |
| 8 | 27729914 | rs77139634 | T | C | 7.52E-05 | 1.2834 |
| 8 | 114910066 | 8:114910066 | A | G | 8.86E-05 | 0.8623 |
| 8 | 114928727 | rs2203099 | A | G | 1.76E-05 | 0.8483 |
| 8 | 114936051 | rs76509867 | A | G | 3.01E-05 | 0.8524 |
| 8 | 114937295 | rs1476309 | C | T | 2.88E-05 | 0.852 |
| 8 | 114939338 | rs2468866 | G | A | 6.04E-05 | 0.8579 |
| 8 | 114939414 | rs2458066 | A | G | 3.99E-05 | 0.8547 |
| 8 | 114940509 | rs1476310 | G | T | 6.50E-05 | 0.8585 |
| 8 | 114940597 | rs1476311 | A | G | 6.50E-05 | 0.8585 |
| 8 | 114941893 | rs74469827 | T | G | 2.01E-05 | 0.8493 |
| 8 | 114944578 | rs75284872 | A | G | 1.97E-05 | 0.8492 |
| 8 | 114945010 | rs12549022 | T | C | 1.85E-05 | 0.8488 |
| 8 | 114945014 | rs12544176 | C | A | 2.66E-05 | 0.8514 |
| 8 | 114945078 | rs16885340 | A | G | 6.34E-05 | 0.8584 |
| 8 | 114949426 | rs74214241 | T | C | 6.78E-05 | 0.8585 |
| 8 | 114950317 | rs2175483 | G | C | 5.60E-05 | 0.8569 |
| 8 | 114950939 | rs1987956 | T | C | 4.55E-05 | 0.8554 |
| 8 | 114952708 | rs58017942 | A | G | 2.23E-05 | 0.85 |
| 8 | 114964723 | rs12682040 | G | A | 2.54E-05 | 0.8541 |
| 8 | 143087167 | rs58603248 | T | A | 1.11E-05 | 1.1989 |
| 8 | 143090966 | rs58473583 | G | A | 2.57E-05 | 1.19 |
| 8 | 143095930 | rs13252175 | C | T | 2.78E-05 | 1.1835 |
| 8 | 143097622 | rs11167111 | G | C | 8.91E-05 | 1.1711 |
| 8 | 143118812 | rs2220696 | C | G | 7.62E-05 | 1.1785 |
| 8 | 143119176 | rs2201177 | T | C | 9.83E-05 | 1.1756 |
| 8 | 143119810 | rs4917295 | A | T | 9.40E-05 | 1.176 |
| 8 | 143120421 | rs13268779 | G | A | 9.82E-05 | 1.1754 |
| 9 | 129636525 | rs11794500 | C | T | 7.15E-09 | 1.3018 |
| 9 | 129638520 | rs72762585 | C | T | 2.55E-09 | 1.3047 |
| 9 | 129665139 | rs13293831 | T | C | 2.51E-08 | 1.2877 |
| 9 | 129673226 | rs592927 | A | G | 4.01E-11 | 1.3399 |
| 10 | 80504270 | rs157981 | C | T | 6.25E-06 | 1.201 |
| 11 | 103979049 | rs12225070 | C | T | 3.88E-06 | 0.8267 |
| 11 | 103979056 | rs12225071 | A | T | 3.88E-06 | 0.8267 |
| 11 | 103979115 | rs12224740 | G | A | 3.88E-06 | 0.8267 |
| 11 | 103979241 | rs12226384 | A | G | 3.30E-06 | 0.8256 |
| 11 | 103980341 | rs11226161 | G | T | 2.56E-06 | 0.8237 |
| 11 | 103980438 | rs11226162 | G | A | 2.56E-06 | 0.8237 |
| 11 | 103980501 | rs11226163 | G | T | 2.56E-06 | 0.8237 |
| 11 | 103980513 | rs11226164 | G | A | 2.56E-06 | 0.8237 |
| 11 | 103980766 | rs11226166 | C | T | 2.36E-06 | 0.8233 |
| 11 | 103980856 | rs10895572 | T | C | 1.97E-06 | 0.8219 |
| 11 | 103981037 | rs10895573 | A | T | 1.97E-06 | 0.8219 |
| 11 | 103981684 | rs11226168 | C | T | 2.16E-06 | 0.8225 |
| 11 | 103981953 | rs12225994 | T | A | 2.16E-06 | 0.8225 |
| 11 | 103982448 | rs11226169 | T | C | 1.70E-06 | 0.8209 |
| 11 | 103982862 | rs10895575 | A | G | 1.62E-06 | 0.8205 |
| 11 | 103984147 | rs1483519 | G | A | 5.90E-05 | 0.847 |
| 11 | 125241046 | rs12273350 | G | A | 1.17E-05 | 1.1847 |
| 12 | 41126732 | rs74896189 | A | G | 3.92E-07 | 1.3738 |
| 13 | 80623346 | rs7981942 | A | C | 2.17E-05 | 1.1852 |
| 13 | 80639977 | rs11149152 | T | C | 5.68E-05 | 1.1612 |
| 13 | 80642677 | rs17072219 | A | C | 1.32E-05 | 1.1761 |
| 16 | 12986742 | rs4781348 | A | C | 1.78E-06 | 1.2067 |
| 20 | 19205830 | rs6035258 | A | G | 1.55E-05 | 0.7803 |
| 20 | 19209214 | rs6075490 | A | G | 8.86E-05 | 0.794 |
| 20 | 19216776 | rs6081544 | A | G | 2.94E-05 | 0.776 |
| 20 | 19220911 | rs3790167 | A | C | 1.68E-05 | 0.7699 |
| 20 | 19224855 | rs3827976 | C | A | 1.75E-05 | 0.7701 |
| 20 | 19225836 | rs62201127 | T | G | 2.15E-05 | 0.7723 |
| 22 | 30819344 | rs117730591 | C | T | 9.98E-08 | 1.3664 |
| 22 | 30947033 | rs116976860 | A | G | 1.18E-07 | 1.3411 |
| 22 | 30992925 | rs57016637 | G | C | 8.36E-05 | 1.2269 |
| 22 | 31007023 | rs75680863 | T | A | 8.77E-06 | 1.2708 |
| 22 | 31041839 | rs9609027 | A | G | 3.48E-05 | 1.2139 |
| 22 | 31186309 | rs3804082 | G | A | 2.43E-06 | 1.2897 |
| 22 | 34222909 | rs12484737 | G | C | 3.66E-05 | 0.8558 |
| 22 | 34229105 | rs5749679 | T | C | 1.14E-07 | 0.8174 |
| 22 | 34235677 | rs5754714 | C | T | 3.39E-07 | 0.8242 |
| 22 | 34236436 | rs5754715 | C | T | 5.88E-07 | 0.8276 |
| 22 | 34237827 | rs5749680 | A | T | 3.87E-07 | 0.8253 |
| 22 | 34238131 | rs5754716 | T | C | 3.58E-07 | 0.8248 |
| 22 | 34238465 | rs738949 | C | G | 2.23E-07 | 0.8221 |
| 22 | 34239559 | rs16993156 | T | A | 3.37E-07 | 0.8243 |
| 22 | 34241811 | rs2051553 | A | G | 2.99E-05 | 1.1667 |
| 22 | 34242570 | rs5754717 | A | G | 5.26E-07 | 0.827 |
| 22 | 34242725 | rs4635624 | G | A | 4.29E-05 | 1.163 |
| 22 | 34245092 | rs2253063 | G | C | 7.82E-07 | 0.8295 |
| 22 | 34245138 | rs2283952 | T | A | 8.57E-07 | 0.83 |
| 22 | 34248314 | rs5749682 | A | C | 7.54E-07 | 0.8291 |
| 22 | 34250366 | rs2018285 | A | G | 9.90E-07 | 0.8266 |
| 22 | 34256108 | rs12159435 | A | T | 4.60E-06 | 0.8369 |
| 22 | 34256369 | rs12159281 | C | A | 2.32E-06 | 0.8322 |
| 22 | 34256853 | rs10854636 | T | C | 2.22E-06 | 0.8318 |
| 22 | 34256860 | rs10854637 | T | C | 1.73E-06 | 0.8302 |
| 22 | 34256908 | rs10854638 | G | A | 1.66E-06 | 0.83 |
| 22 | 34256919 | rs10854639 | C | T | 1.72E-06 | 0.8302 |
| 22 | 34256923 | rs10854640 | A | C | 1.56E-06 | 0.8296 |
| 22 | 34257133 | rs17809399 | A | G | 2.28E-06 | 0.8321 |

**Table S3. Expression quantitative trait loci （eQTL）analysis of GWAS risk variants**

| **SNP** | **Target gene** | **CMC（*P*-value）** | **LIBD（*P*-value）** |
| --- | --- | --- | --- |
| rs1801133 | *MFN2* | 2.69×10^-03^ | NA |
| rs1801133 | *MTHFR* | 5.83×10^-03^ | NA |
| rs1801133 | *NPPA-AS1* | NA | 3.63×10^-06^ |
| rs592927 | *ANGPTL2* | 2.99×10^-03^ | NA |
| rs592927 | *RALGPS1* | 4.01×10^-06^ | NA |
| rs7626288 | *TDGF1* | NA | 5.67×10^-05^ |

**Table S4. eQTL and meQTL of *MTHFR* that show association with EOS**

| **SNP** | **Type** | **P_QTL_** | **P_EOS_** |
| --- | --- | --- | --- |
| rs1801133 | eQTL | 0.0058 | 7.48E-05 |
| rs2981953 | eQTL | 0.0011 | 0.0019 |
| rs12402363 | eQTL | 0.00072 | 0.010 |
| rs12406383 | eQTL | 0.00077 | 0.010 |
| rs198369 | eQTL | 0.0011 | 0.027 |
| rs1801133 | meQTL | 1.67E-12 | 7.48E-05 |
| rs2981953 | meQTL | 7.14E-13 | 0.0019 |
| rs198389 | meQTL | 3.95E-12 | 0.0020 |
| rs198379 | meQTL | 4.45E-12 | 0.0024 |
| rs549596 | meQTL | 3.82E-14 | 0.0056 |
| rs198388 | meQTL | 5.14E-14 | 0.021 |
| rs198369 | meQTL | 3.74E-16 | 0.027 |
| rs198358 | meQTL | 7.70E-09 | 0.033 |

**Table S5. MAGMA GO/KEGG enrichment analysis of EOS GWAS summary statistics（top10 is shown）**

| **GO_NAME** | ***P* value** |
| --- | --- |
| GO_NEGATIVE_REGULATION_OF_AXON_EXTENSION | 0.00013 |
| GO_POSITIVE_REGULATION_OF_LEUKOCYTE_CHEMOTAXIS | 0.00023 |
| GO_NEGATIVE_REGULATION_OF_GLIOGENESIS | 0.00028 |
| GO_POSITIVE_REGULATION_OF_CHEMOTAXIS | 0.00068 |
| GO_NEGATIVE_REGULATION_OF_GLIAL_CELL_PROLIFERATION | 0.00069 |
| GO_REGULATION_OF_LEUKOCYTE_CHEMOTAXIS | 0.00082 |
| GO_ACETYL_COA_BIOSYNTHETIC_PROCESS_FROM_PYRUVATE | 0.00092 |
| GO_REGULATION_OF_MONONUCLEAR_CELL_MIGRATION | 0.00092 |
| GO_REGULATION_OF_DEVELOPMENT_HETEROCHRONIC | 0.0010 |
| GO_MAINTENANCE_OF_CELL_NUMBER | 0.0012 |

**Table S6. Functional annotation of the index snps of four EOS GWS loci by RegulomeDB**

| **Chr^a^** | **Position** | **SNP** | **Rank^b^** | **Score^c^** | **ChIP data^d^** | **Strong transcription^e^** | | **Enhancer^f^** | **Accessibility^g^** | |
| --- | --- | --- | --- | --- | --- | --- | --- | --- | --- | --- |
| 1 | 11856378 | rs1801133 | 4 | 0.61 | 92 | 104 | 21 | | | 18 |
| 1 | 82919925 | rs1281571 | 7 | 0.18 | 0 | 0 | 1 | | | 0 |
| 3 | 46485369 | rs7626288 | 5 | 0.13 | 0 | 12 | 3 | | | 7 |
| 9 | 129673226 | rs592927 | 6 | 0.00 | 0 | 1 | 0 | | | 0 |

^a^ Chromosome ; ^b^ RegulomeDB rank score, detailed about the Rank score representation please check this link for detail (<https://www.regulomedb.org/regulome-help/>); ^c^ RegulomeDB probability score which ranges from 0-1, The higher score indicating the higher probability of a SNP likely to be a regulatory SNP; ^d^ The number of ChIP-seq experiments peak signals that overlap with the SNP; ^e^ the number of Chromatin state datasets (for study transcription signal) that overlap the SNP. ^f^ the number of Chromatin state dataset (for study enhancer signal) that overlap the SNP. ^g^ the number of the FAIRE-seq and DNase-seq experiments signals that overlap with SNP.

**Table S7. The association significance of genome wide significant risk variants (from a recent large-scale trans-ancestry meta-analysis of East Asians and PGC2 Europeans) with EOS in our discovery stage**

| **chr** | **position** | **snp** | **A1** | **A2** | **P** | **OR** |
| --- | --- | --- | --- | --- | --- | --- |
| 9 | 129705507 | rs13284900 | T | C | 0.0005459 | 1.2411 |
| 2 | 200715388 | rs2949006 | T | G | 0.0008679 | 1.2215 |
| 1 | 243690491 | rs13376709 | T | C | 0.003539 | 1.1638 |
| 11 | 46711854 | rs7926389 | G | A | 0.01401 | 1.224 |
| 4 | 176859992 | rs2333321 | A | G | 0.01481 | 1.2168 |
| 2 | 28024984 | rs4632296 | T | C | 0.02373 | 1.13 |
| 14 | 71388398 | rs2189806 | A | T | 0.02408 | 1.1271 |
| 18 | 77622879 | rs28735056 | G | A | 0.03739 | 1.1134 |
| 10 | 104793648 | rs75970938 | C | T | 0.03898 | 0.885 |
| 16 | 64373894 | rs2010476 | G | C | 0.03908 | 0.8946 |
| 11 | 133827733 | rs3802924 | C | A | 0.04853 | 1.2824 |
| 7 | 86420126 | rs13230421 | A | T | 0.04954 | 1.2568 |
| 7 | 24717969 | rs79210963 | C | T | 0.05218 | 1.2731 |
| 8 | 143316970 | rs13262595 | A | G | 0.05535 | 1.1185 |
| 15 | 83254708 | rs783540 | G | A | 0.05682 | 1.1039 |
| 1 | 36275112 | rs12083902 | G | A | 0.05774 | 1.1183 |
| 4 | 118827751 | rs28419375 | C | T | 0.06609 | 1.1114 |
| 3 | 63842629 | rs832190 | T | C | 0.06626 | 0.9079 |
| 14 | 72416219 | rs2190864 | T | C | 0.06747 | 1.111 |
| 10 | 64862477 | rs112222723 | C | T | 0.06988 | 1.1239 |
| 19 | 2686461 | rs11878180 | C | A | 0.07491 | 0.9077 |
| 1 | 44100084 | rs11210892 | G | A | 0.0763 | 1.1033 |
| 17 | 55728224 | rs4793885 | A | G | 0.07975 | 1.0975 |
| 4 | 24269622 | rs4697446 | G | T | 0.08143 | 1.0958 |
| 3 | 36871993 | rs3732386 | T | C | 0.088 | 0.7891 |
| 3 | 161780488 | rs6804239 | C | T | 0.09156 | 1.0929 |
| 8 | 4183057 | rs13261217 | G | A | 0.09513 | 0.9078 |
| 16 | 4469284 | rs13335685 | T | C | 0.1046 | 1.0976 |
| 17 | 17912548 | rs4365348 | C | T | 0.1105 | 0.867 |
| 11 | 57510294 | rs9420 | A | G | 0.1123 | 1.1159 |
| 20 | 48129169 | rs7271624 | G | C | 0.1283 | 0.9064 |
| 2 | 60785937 | rs12328348 | G | A | 0.136 | 0.8952 |
| 2 | 198375329 | rs7605813 | C | G | 0.1372 | 0.9247 |
| 1 | 173646773 | rs61826793 | A | G | 0.1503 | 1.0886 |
| 8 | 18442351 | rs2634447 | C | T | 0.1692 | 1.0981 |
| 12 | 108609634 | rs10861879 | G | A | 0.1796 | 0.9328 |
| 15 | 44257954 | rs2957582 | T | C | 0.1815 | 0.9283 |
| 3 | 180838644 | rs4855019 | T | C | 0.1915 | 0.8962 |
| 2 | 58383820 | rs7596038 | T | C | 0.193 | 0.9338 |
| 7 | 21534152 | rs7811417 | T | C | 0.1978 | 1.0697 |
| 12 | 110723245 | rs4766428 | C | T | 0.1996 | 0.8881 |
| 15 | 91426560 | rs4702 | G | A | 0.2041 | 1.069 |
| 7 | 94267862 | rs1357318 | C | T | 0.2117 | 0.9305 |
| 7 | 2029867 | rs58120505 | T | C | 0.2147 | 1.0684 |
| 7 | 104930250 | rs2057884 | T | C | 0.219 | 1.0694 |
| 12 | 29928388 | rs302321 | A | C | 0.226 | 0.9296 |
| 1 | 177280121 | rs6670165 | T | C | 0.2472 | 1.0849 |
| 16 | 30000901 | rs3814881 | A | G | 0.251 | 1.0645 |
| 10 | 18725659 | rs1926026 | G | A | 0.2614 | 0.8699 |
| 11 | 45330221 | rs11038391 | T | G | 0.271 | 0.9436 |
| 5 | 60614879 | rs4132385 | T | G | 0.2837 | 1.0728 |
| 5 | 153685526 | rs6864084 | T | C | 0.2967 | 1.261 |
| 15 | 89900887 | rs758129 | A | G | 0.3081 | 1.0643 |
| 11 | 24374545 | rs12363019 | A | T | 0.3147 | 1.0838 |
| 8 | 60694647 | rs867743 | G | A | 0.3173 | 1.055 |
| 12 | 122583781 | rs147786161 | G | A | 0.3217 | 0.95 |
| 10 | 53940526 | rs61847303 | T | C | 0.3304 | 0.9195 |
| 11 | 130718813 | rs10791099 | G | C | 0.3361 | 0.9512 |
| 2 | 146440672 | rs2381760 | G | A | 0.3402 | 1.0878 |
| 12 | 2346393 | rs2159100 | T | C | 0.3469 | 1.1101 |
| 6 | 43185733 | rs6938026 | G | A | 0.3524 | 0.9519 |
| 1 | 8495590 | rs172531 | G | A | 0.3533 | 0.9108 |
| 20 | 37366737 | rs6128857 | T | A | 0.3586 | 0.9485 |
| 13 | 56948260 | rs71428218 | T | C | 0.3669 | 0.9406 |
| 12 | 121627268 | rs11615992 | G | A | 0.3843 | 1.0559 |
| 5 | 152601914 | rs2927176 | A | T | 0.3981 | 0.9548 |
| 1 | 2379705 | rs942820 | A | G | 0.4106 | 0.9281 |
| 7 | 137070298 | rs7785663 | A | G | 0.423 | 1.0472 |
| 2 | 225391296 | rs11685299 | A | C | 0.439 | 1.0469 |
| 9 | 84799537 | rs2767713 | A | C | 0.4656 | 0.9603 |
| 11 | 124613957 | rs55661361 | A | G | 0.4668 | 0.9586 |
| 5 | 109012310 | rs419677 | A | G | 0.4757 | 1.0407 |
| 13 | 32763757 | rs9567393 | G | A | 0.4906 | 0.9626 |
| 21 | 16439883 | rs9975024 | G | A | 0.4937 | 1.0369 |
| 16 | 9945919 | rs41356645 | T | C | 0.5045 | 1.0658 |
| 6 | 105466332 | rs160593 | G | A | 0.5077 | 0.9626 |
| 3 | 30304206 | rs6797774 | T | G | 0.5135 | 1.0349 |
| 2 | 193927136 | rs13019859 | A | G | 0.5155 | 1.0351 |
| 2 | 134842307 | rs6721549 | C | T | 0.5164 | 1.0346 |
| 22 | 39967430 | rs5757730 | A | G | 0.5502 | 1.082 |
| 15 | 61856263 | rs35225048 | C | T | 0.5508 | 1.0344 |
| 8 | 10226355 | rs3088186 | T | C | 0.5531 | 1.0387 |
| 5 | 45305615 | rs6874127 | G | A | 0.5673 | 0.9708 |
| 5 | 137840860 | rs13164092 | G | A | 0.5682 | 0.964 |
| 8 | 111506565 | rs6995307 | G | C | 0.5823 | 0.9607 |
| 19 | 50148052 | rs12104272 | A | G | 0.5869 | 1.032 |
| 1 | 98552832 | rs1198588 | A | T | 0.5975 | 1.0538 |
| 2 | 104984387 | rs62152284 | T | C | 0.6077 | 1.028 |
| 10 | 3819714 | rs1043009 | C | T | 0.608 | 1.0272 |
| 6 | 108988184 | rs2153960 | G | A | 0.6094 | 1.0307 |
| 11 | 123394636 | rs77502336 | C | G | 0.6153 | 1.0266 |
| 1 | 30437268 | rs6694545 | A | G | 0.6252 | 1.029 |
| 12 | 117708611 | rs28607014 | T | C | 0.6582 | 0.9731 |
| 12 | 92257133 | rs10777342 | T | C | 0.707 | 0.9782 |
| 11 | 109378071 | rs12421382 | T | C | 0.711 | 0.9779 |
| 7 | 78337700 | rs323169 | C | T | 0.7159 | 1.026 |
| 12 | 123682081 | rs1716180 | G | A | 0.7315 | 0.9808 |
| 2 | 172833139 | rs13004237 | A | G | 0.7352 | 1.0203 |
| 4 | 103937933 | rs7676765 | T | C | 0.736 | 0.9827 |
| 4 | 23423586 | rs215412 | A | G | 0.7431 | 0.9673 |
| 15 | 85124642 | rs11633534 | T | C | 0.7506 | 0.9766 |
| 1 | 111322019 | rs1999512 | G | A | 0.7575 | 1.0162 |
| 3 | 52628056 | rs2577831 | A | C | 0.7592 | 0.9842 |
| 4 | 170303984 | rs57346805 | C | T | 0.7799 | 0.9827 |
| 19 | 36530913 | rs3810449 | T | C | 0.7856 | 0.9831 |
| 7 | 71852173 | rs79527984 | A | G | 0.7885 | 0.9848 |
| 11 | 113392994 | rs2514218 | T | C | 0.7916 | 0.959 |
| 2 | 185663304 | rs4666990 | T | C | 0.8137 | 0.9879 |
| 7 | 110867535 | rs214475 | G | A | 0.8273 | 1.0113 |
| 8 | 26242272 | rs117325001 | T | G | 0.8322 | 1.0113 |
| 7 | 110074276 | rs9656169 | T | C | 0.8377 | 0.9867 |
| 15 | 70589272 | rs12148337 | C | T | 0.8459 | 0.9898 |
| 3 | 76469374 | rs3849490 | T | C | 0.8762 | 1.0082 |
| 2 | 233593499 | rs17164 | C | T | 0.8967 | 0.9896 |
| 18 | 69774278 | rs337718 | T | C | 0.8987 | 0.9928 |
| 1 | 97173906 | rs12031518 | C | T | 0.9373 | 0.995 |
| 6 | 84401807 | rs217289 | A | G | 0.9672 | 0.9979 |
| 14 | 60165070 | rs4243617 | T | G | 0.9715 | 1.0019 |
| 18 | 27501492 | rs1440849 | G | A | 0.9753 | 0.9981 |
| 8 | 17036201 | rs17687067 | C | A | 0.9804 | 1.0014 |
| 16 | 13748916 | rs7499362 | G | C | 0.983 | 1.0019 |
| 11 | 79230921 | rs7951609 | C | T | 0.9969 | 0.9998 |
| 13 | 58702746 | rs9569820 | T | G | 0.9974 | 1.0002 |
| 8 | 103669991 | rs4734654 | G | A | 0.9993 | 1.0001 |

**References:**

1. Fromer, M. et al. Gene expression elucidates functional impact of polygenic risk for schizophrenia. *Nat Neurosci*. **19**, 1442-1453 (2016).

2. Huang, T. & Cai, Y. D. An information-theoretic machine learning approach to expression QTL analysis. *PLoS One*. **8**, e67899 (2013).

3. Jaffe, A. E. et al. Developmental and genetic regulation of the human cortex transcriptome illuminate schizophrenia pathogenesis. *Nat Neurosci*. **21**, 1117-1125 (2018).

4. Wang, D. et al. Comprehensive functional genomic resource and integrative model for the human brain. *Science*. **362**, (2018).

5. Hannon, E. et al. Methylation QTLs in the developing brain and their enrichment in schizophrenia risk loci. *Nat Neurosci*. **19**, 48-54 (2016).

6. Pardinas, A. F. et al. Common schizophrenia alleles are enriched in mutation-intolerant genes and in regions under strong background selection. *Nat Genet*. **50**, 381-389 (2018).

7. Li, Z. et al. Genome-wide association analysis identifies 30 new susceptibility loci for schizophrenia. *Nat Genet*. **49**, 1576-1583 (2017).

8. Lam, M. et al. Comparative genetic architectures of schizophrenia in East Asian and European populations. *Nat Genet*. **51**, 1670-1678 (2019).

9. Lee, S. H., Goddard, M. E., Wray, N. R. & Visscher, P. M. A better coefficient of determination for genetic profile analysis. *Genet Epidemiol*. **36**, 214-224 (2012).

10. Bulik-Sullivan, B. K. et al. LD Score regression distinguishes confounding from polygenicity in genome-wide association studies. *Nat Genet*. **47**, 291-295 (2015).

11. de Leeuw, C. A., Mooij, J. M., Heskes, T. & Posthuma, D. MAGMA: generalized gene-set analysis of GWAS data. *PLoS Comput Biol*. **11**, e1004219 (2015).

12. Watanabe, K., Taskesen, E., van Bochoven, A. & Posthuma, D. Functional mapping and annotation of genetic associations with FUMA. *Nat Commun*. **8**, 1826 (2017).
